# The universe of galectin-binding partners and their functions in health and disease

**DOI:** 10.1016/j.jbc.2023.105400

**Published:** 2023-10-26

**Authors:** María F. Troncoso, María T. Elola, Ada G. Blidner, Luciana Sarrias, María V. Espelt, Gabriel A. Rabinovich

**Affiliations:** 1 Departamento de Química Biológica, Facultad de Farmacia y Bioquímica, Universidad de Buenos Aires, Buenos Aires, Argentina; 2 Instituto de Química y Fisicoquímica Biológicas (IQUIFIB) Prof Alejandro C. Paladini, CONICET−Universidad de Buenos Aires, Buenos Aires, Argentina; 3 Laboratorio de Glicomedicina, Instituto de Biología y Medicina Experimental (IBYME-CONICET), Buenos Aires, Argentina; 4 Facultad de Ciencias Exactas y Naturales, Universidad de Buenos Aires, Buenos Aires, Argentina

**Keywords:** galectins, receptors, glycosylation, glycoproteins, immunity, angiogenesis, tumorigenesis

## Abstract

Galectins, a family of evolutionarily conserved glycan-binding proteins, play key roles in diverse biological processes including tissue repair, adipogenesis, immune cell homeostasis, angiogenesis, and pathogen recognition. Dysregulation of galectins and their ligands has been observed in a wide range of pathologic conditions including cancer, autoimmune inflammation, infection, fibrosis, and metabolic disorders. Through protein–glycan or protein–protein interactions, these endogenous lectins can shape the initiation, perpetuation, and resolution of these processes, suggesting their potential roles in disease monitoring and treatment. However, despite considerable progress, a full understanding of the biology and therapeutic potential of galectins has not been reached due to their diversity, multiplicity of cell targets, and receptor promiscuity. In this article, we discuss the multiple galectin-binding partners present in different cell types, focusing on their contributions to selected physiologic and pathologic settings. Understanding the molecular bases of galectin–ligand interactions, particularly their glycan-dependency, the biochemical nature of selected receptors, and underlying signaling events, might contribute to designing rational therapeutic strategies to control a broad range of pathologic conditions.

Galectins are a family of evolutionary conserved glycan-binding proteins that recognize multiple *N*-acetyllactosamine (LacNAc, Galβ1,4GlcNAc) units present in *N*-and *O*-glycans on cell surface glycoconjugates. These lectins are defined by at least one carbohydrate recognition domain (CRD) with affinity for β-galactosides and conserved sequence motifs (1). To date, 15 galectins have been described in mammals and according to their structural features, they are classified into three groups: “proto-type” galectins (galectin-1, galectin-2, galectin-5, galectin-7, galectin-10, galectin-11/-15, galectin-13, galectin-14, and galectin-16) contain one CRD and can dimerize; “tandem repeat-type” galectins (galectin-4, galectin-6, galectin-8, galectin-9, and galectin-12) contain two distinct CRD in tandem, connected by a linker peptide; and “chimera-type” galectin-3 which consists of unusual proline- and glycine-rich short stretches fused onto the CRD (1) (Fig. 1). In particular, galectin-1, -2, -3, -4, -7, -8, -9, -10, -12, -13, -14 and -16 have been identified in human tissues.

**Figure 1:**
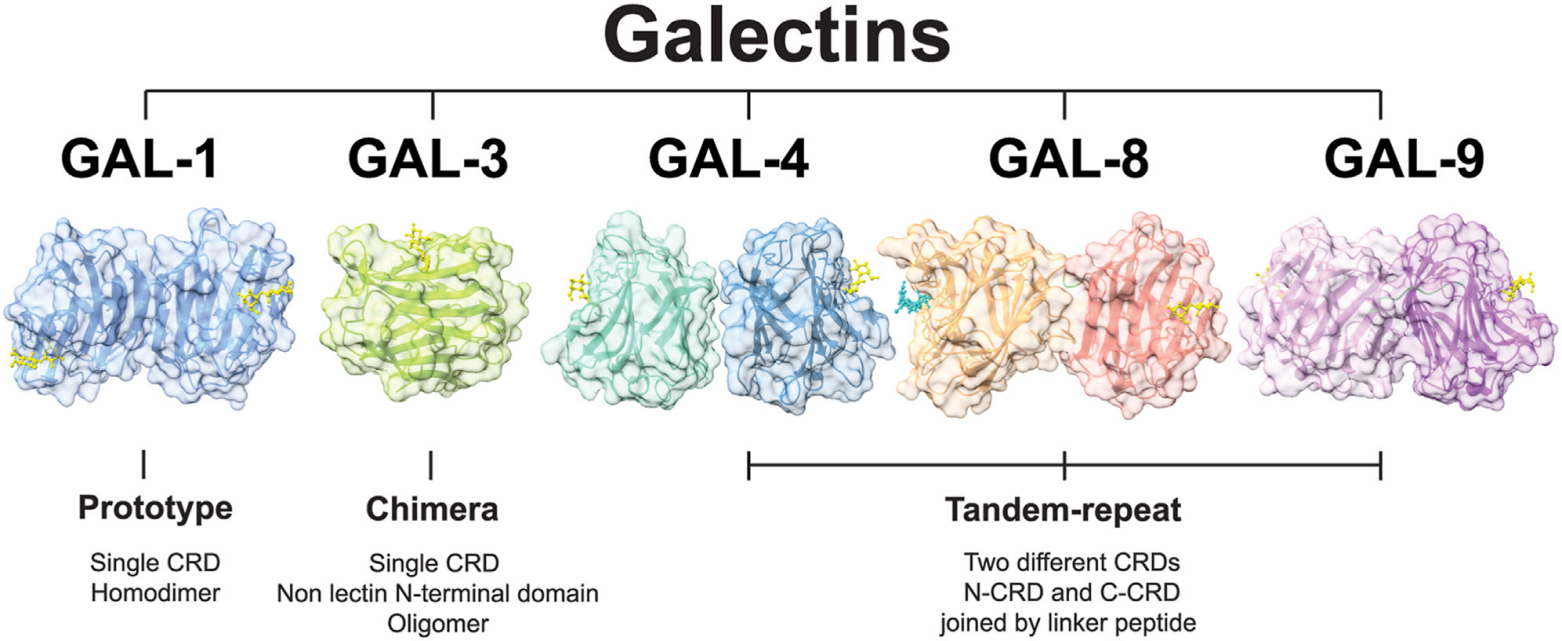
**Conserved structuresof selected members of the galectin family and structural classification.** Based on their structural features, galectins are classified into three groups: “proto-type” galectins (*e.g.* GAL-1) contain one carbohydrate recognition domain (CRD) and can dimerize; “tandem repeat-type” galectins (*e.g.* GAL-4, GAL-8 and GAL-9) contain two distinct CRD in tandem, connected by a linker peptide; and “chimera-type” GAL-3 which consists of unusual proline- and glycine-rich short stretches fused onto the CRD. GAL-1 structure is shown in *blue* (PDB: 4Y1U); GAL-3 structure in *green* (PDB: 4R9A); GAL-4 N-CRD in *turquoise* (PDB: 5DUV) and GAL-4 C-CRD in *steel blue* (PDB: 4YM3); protease-resistant mutant GAL-8 form possessing both N-CRD (shown in *orange*), and C-CRD (shown in *red*), with a linker of two amino acids (His-Met) (shown in *green*) (PDB: 3VKM); protease-resistant mutant GAL-9 form possessing both N-CRD (shown in *lilac*) and C-CRD (shown in *violet*) with a linker of 19 amino acids (shown in *green*) and a metal ion found at the CRDs interface (PDB: 3WV6). All structures are represented in complex with lactose in *yellow* and GAL-8 N-CRD with SiaLac in *cyan*.

Interestingly, galectin CRDs can differ in amino acid sequences outside conserved sites, thus providing the ability to recognize diverse glycan structures (Figure 2, Figure 3, Figure 4, Figure 5) (2). Furthermore, modifications on LacNAc or poly-LacNAc structures may alter galectin-glycan interactions. For instance, some galectins can recognize addition of a terminal sialic acid or internal fucose in the LacNAc sequence, while others may lose their ability to bind LacNAc in the presence of such modifications (2, 3). In fact, incorporation of α2-6-linked sialic acid to cell surface glycoconjugates interrupts binding of some members of the family, including galectin-1. Moreover, galectins may differ in their ability to recognize LacNAc in a terminal position or internal repetitions within the glycan structure. This relative selectivity may explain the functional variations observed among individual galectin family members, and even between N- and C-CRDs in “tandem repeat-type” galectins (Figure 2, Figure 3, Figure 4, Figure 5) (4, 5, 6). The affinity of galectins for *N*-glycans also increases in correlation with β1-6 branching, mediated by the β1,6-*N*-acetylglucosaminyltransferase V (MGAT5/GnTV) and extension with poly-*N*-acetyllactosamine (4). Therefore, the cellular response triggered by interaction of a given galectin and its binding partner will depend not only on the specific galectin family member and its preferred ligand but also on the expression and activity of glycosyltransferases and/or glycohydrolases in a particular cellular context. For instance, important changes in glycosylation occurring during tumorigenesis, metastasis, and inflammation, including an increase in *N*-glycan branching, may affect galectin binding to cancer-associated glycoproteins (7, 8).

**Figure 2:**
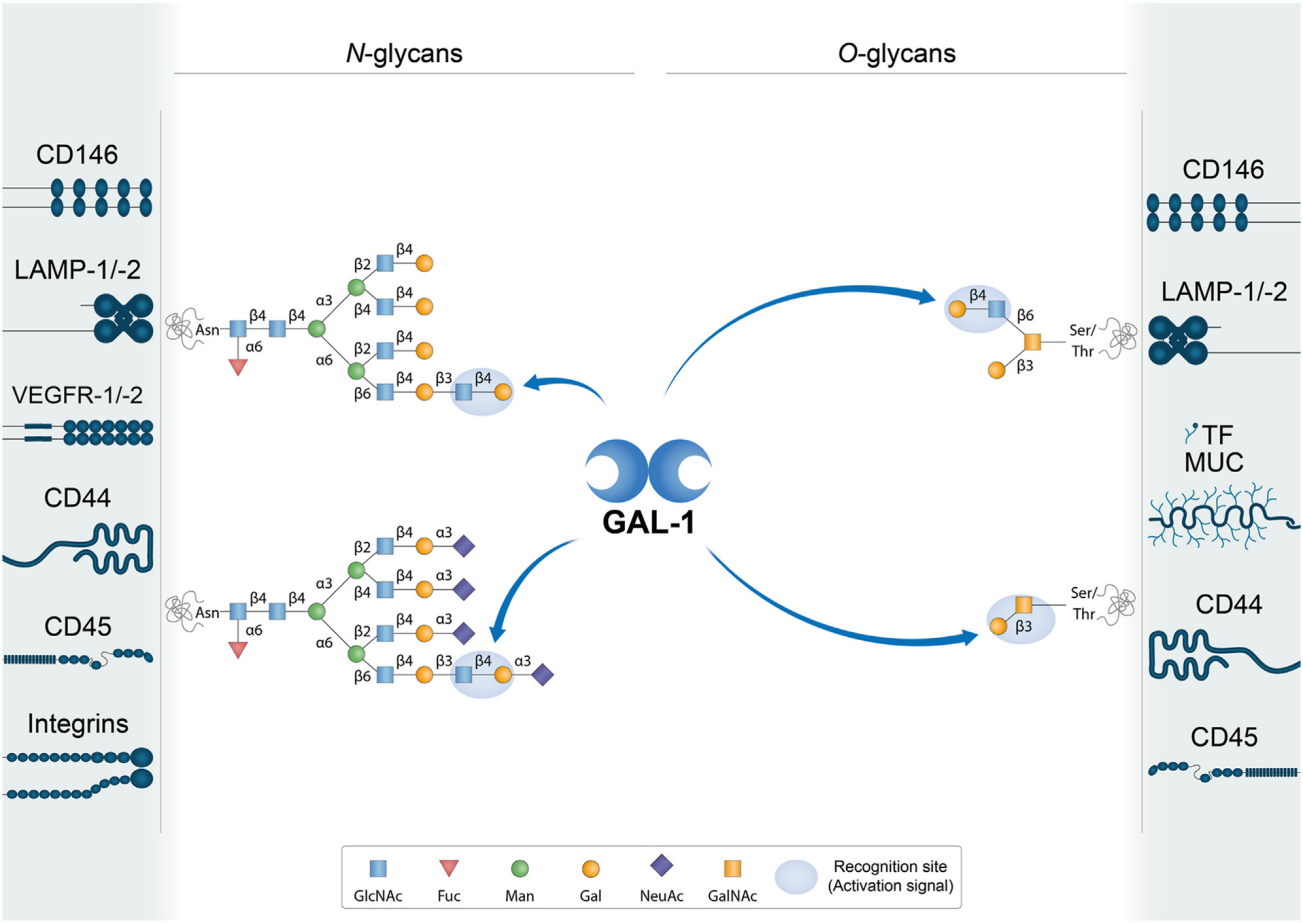
**Schematic representation of potential GAL-1-glycan interactions on selected cell surface receptors.** GAL-1 preferentially recognizes terminal LacNAc residues on both *N*- and *O*-glycans. It can bind to terminal α2-3-linked sialic acid in the LacNAc sequence, but α2-6-linked sialic acid prevents the binding of this lectin. LacNAc: Galβ1-4GlcNAc. Glycans are represented according to the symbol nomenclature proposed (244).

**Figure 3:**
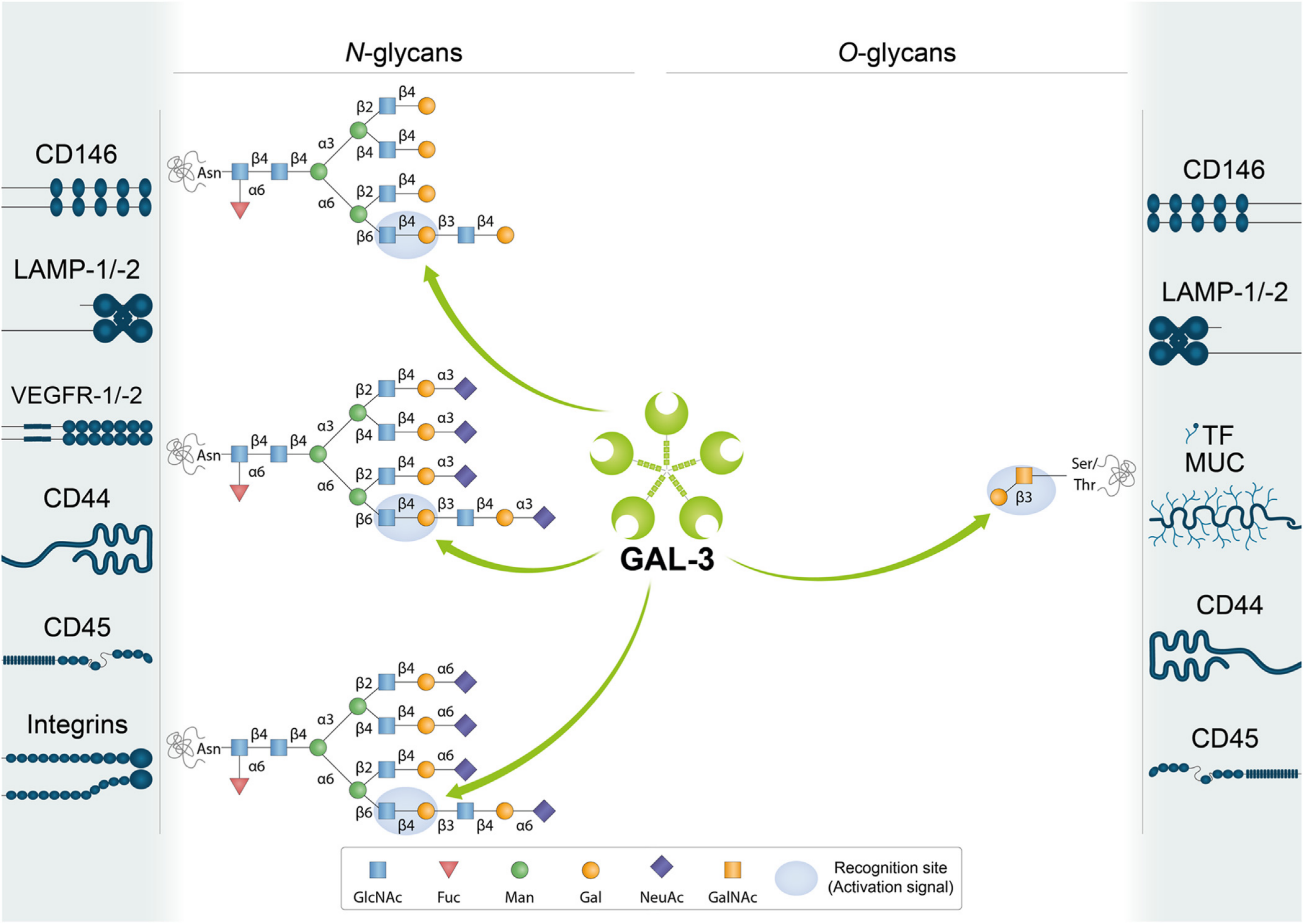
**Schematic representation of potential GAL-3-glycan interactions on selected cell surface receptors.** GAL-3 preferentially binds to internal LacNAc residues on both *N*- and *O*-glycans. Terminal α2-3-linked or α2-6-linked sialic acid in the LacNAc sequence is permissive for binding of GAL-3, which can interact with internal LacNAc repeats. LacNAc: Galβ1-4GlcNAc. Glycans are represented according to the symbol nomenclature proposed (244).

**Figure 4:**
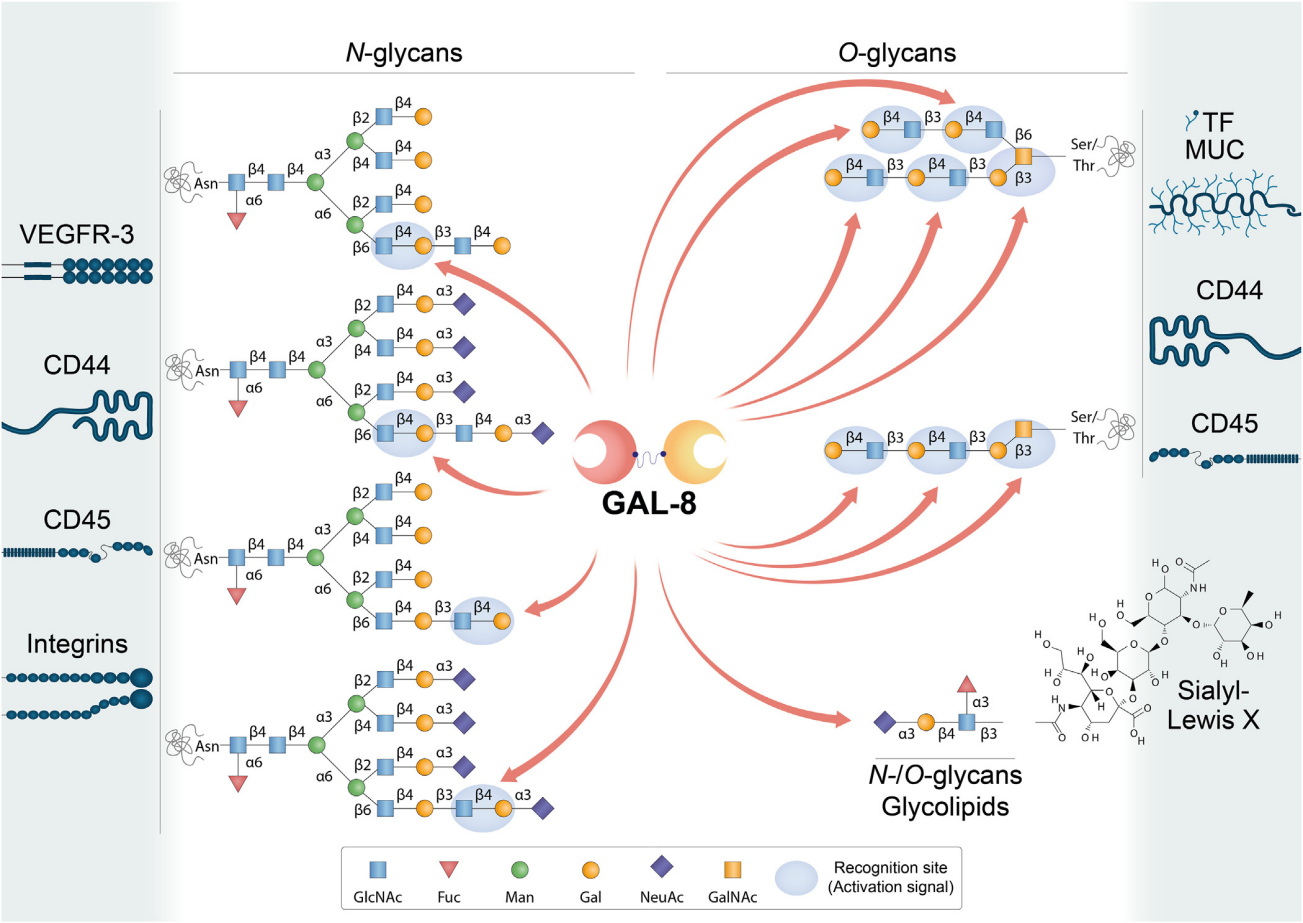
**Schematic representation of potential GAL-8-glycan interactions on selected cell surface receptors.** GAL-8 binds to *N*- and *O*-glycans, and glycolipids. It preferentially recognizes internal -but also terminal- LacNAc residues. Sialyl-Lewis X tetrasaccharide (Neu5Acα2-3Galβ1-4[Fucα1-3]GlcNAcβ) may interact with GAL-8 in *N*- and *O*-glycans as well as in glycolipids. The N-terminal CRD of GAL-8 (GAL-8N) has a high binding affinity for α2-3-sialylated- or 3-sulfated β-galactosides and sialyl-Lewis X-containing *N*- or *O*-glycans and glycolipids. LacNAc: Galβ1-4GlcNAc. 3′Sialyl-LacNAc: Neu5Acα2-3Galβ1-4GlcNAc. 3′Sulfo-LacNAc: Sulfo-3Galβ1-4GlcNAc. Glycans are represented according to the symbol nomenclature proposed (244).

**Figure 5:**
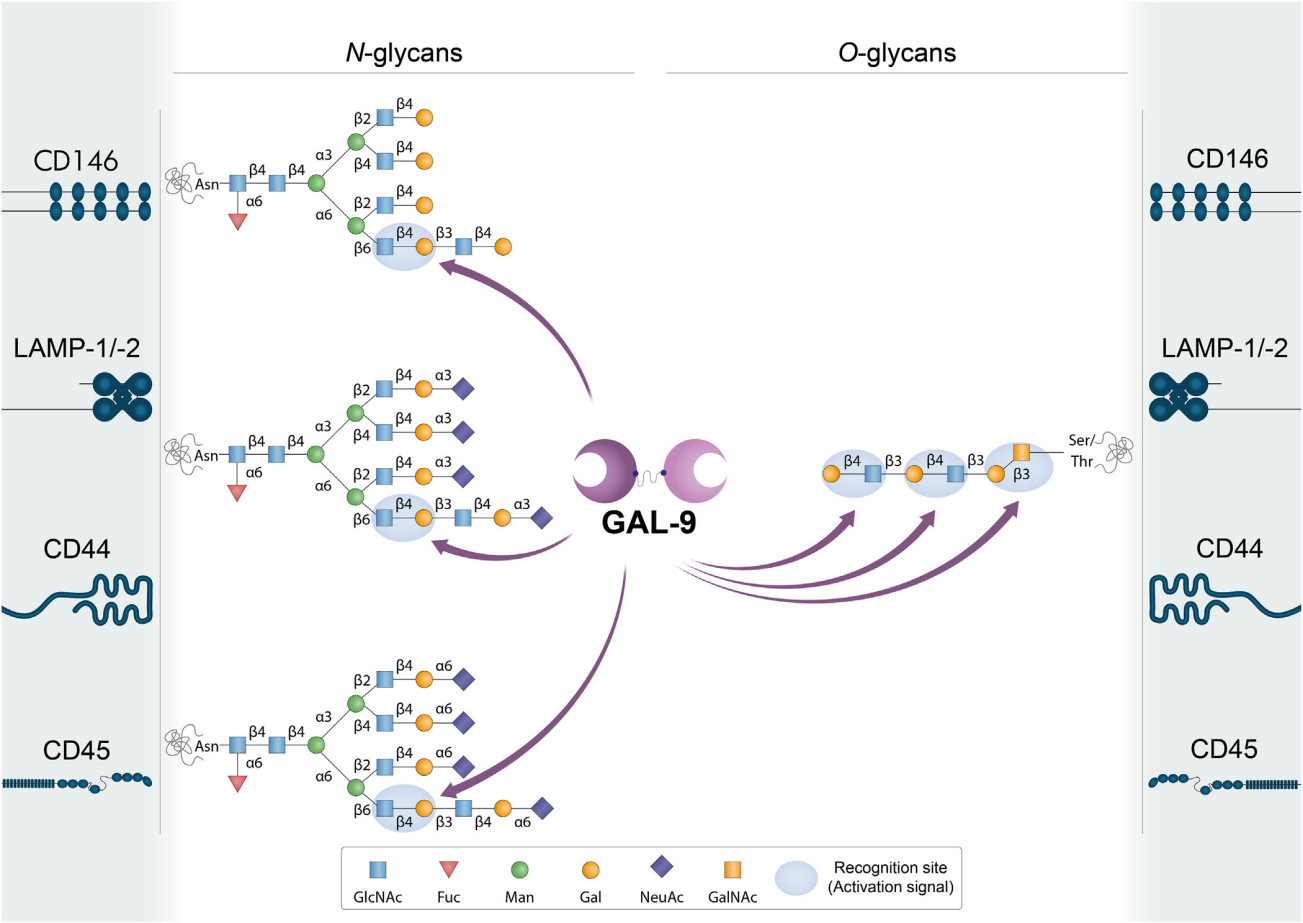
**Schematic representation of GAL-9-glycan interactions on selected cell surface receptors.** GAL-9 binds to *N*- and *O*-glycans. It preferentially recognizes internal LacNAc residues. The N-terminal CRD of GAL-9 (GAL-9N) has a high affinity for gangliosides (*e.g*. GA1, GM1, GD1a) and other glycolipids (ended in *e.g.* Förssman pentasaccharide, A-hexasaccharide). LacNAc: Galβ1-4GlcNAc. Glycans are represented according to the symbol nomenclature proposed (244).

The interaction between galectins and their specific ligands may lead to a broad range of biological activities that are dependent on the cell type and tissue context, the expression levels of individual galectins, and the potential receptor/s involved. While some members of the family (*e.g.* galectin-1, -3, and -9) are widely expressed across different cells, including, immune, endothelial, and epithelial cells, as well as sensory neurons, others have a more restricted tissue localization, including galectin-7 that is mainly localized in the skin, galectin-10 that is strongly represented in eosinophils and basophils, and galectin-12, a protein abundantly expressed in adipose tissue (9). Notably, the biological activity of galectins can be influenced by their dimerization or oligomerization state, selective exposure of *N*- and *O*-glycans on target cells, and the redox status of different tissue microenvironments (10).

Although most galectins are secreted to the extracellular medium, they do not possess the signal peptide required for export *via* the classical secretory pathway (11). While the underlying mechanism of secretion still remains poorly understood, it appears to involve the accumulation of galectins in discrete patches beneath the plasma membrane and externalization through extracellular vesicles (11). Recent findings revealed the contribution of inflammatory cell death pathways, including pyroptosis and necroptosis (12) as well as autophagy (13) to galectin externalization. Once in the extracellular medium, galectins can form multivalent complexes with cell surface glycoconjugates, often termed lattices, and transduce intracellular signals, leading to modulation of a wide range of cellular processes including proliferation, differentiation, and survival (14, 15, 16). Furthermore, galectins can also control intracellular processes, including mRNA splicing, cell cycle progression, apoptosis, and autophagy through protein–protein or protein–glycan interactions (17, 18, 19, 20).

Galectins are critical regulators of a broad range of physiological processes, such as acute and chronic inflammation (21, 22), pathogen recognition (23, 24) and pregnancy (25), among others. Mounting evidence indicates that these proteins also play fundamental roles in cancer biology including tumor cell proliferation, angiogenesis, migration, metastasis and immune escape. Given these pleiotropic activities in the tumor microenvironment and their emerging roles in resistance to several cancer therapies, galectins are currently being considered as novel targets in oncology (26, 27, 28, 29).

In this article, we focus on the diversity of receptors and binding partners that galectins can engage on different cell types and the extracellular matrix (ECM), the glycan dependency and multivalent nature of these interactions, and the underlying signaling pathways and cellular functions elicited by galectin-receptor signaling complexes in physiologic and pathologic settings.

## Galectins and their binding partners

Galectins bind to a discrete set of glycosylated receptors expressed on the surface of a wide variety of cell types (Table 1, Table 2, Table 3, Table 4, Table 5) through recognition of β-galactoside-containing *N*- and *O*-glycans (Figure 2, Figure 3, Figure 4, Figure 5) (30, 31). In this section, we review current knowledge on different glycosylated receptors engaged by individual members of the galectin family, particularly galectin (GAL)-1, -3, -4, -8 and -9 on endothelial, immune, and tumor cells and discuss the biochemical nature of these interactions.

**Table 1:** GAL-1-binding partners on different cell types

GAL-1-binding partner	Cell type/tissue	Function	Localization	References
NRP1	Neurons	Axonal regeneration	Cell surface	(36)
	Endothelial cells	Angiogenesis, cell permeability	Cell surface	(37)
	Hepatic stellate cells	Fibrosis	Cell surface	(41)
	Gingival fibroblasts	Cutaneous wound healing	Cell surface	(42)
VEGFR-1/VEGFR-2	Endothelial cells	Angiogenesis, cell permeability	Cell surface	(43, 44, 45)
TF/MUC1	Breast cancer cells	Cancer cell adhesion to endothelium	Cell surface	(53)
	Trophoblasts, endometrial cells, oocytes, chorioncarcinoma, endometrioid adenocarcinoma	Cell proliferation, implantation, maintenance of pregnancy	Cell surface	(54, 55, 56)
CD146	Endothelial cells	Apoptotic regulation	Cell surface	(48)
	Brown adipose tissue	Chronic inflammation	Cell surface	(49)
LAMP-1/-2	CHO, ovarian and colon carcinoma cells	Cell adhesion to ECM proteins	Cell surface	(66, 67, 68)
	Secretory granules released by cytotoxic T cells	Regulation of cytotoxic T cell activity		(69)
CEA	Colon carcinoma cells	Cell adhesion, migration, metastasis	Cell surface	(70, 74)
Integrins (α_1_β_1_, α_5_β_1_, α_7_β_1_, α_2_, α_3_, α_v_α_IIb_β_3_)	Myoblasts, skeletal and vascular smooth muscle cells	Cell adhesion to ECM proteins, muscle cell differentiation	Cell surface	(80, 81, 82)
	Trophoblasts	Trophoblast cell adhesion to ECM, invasion, migration	Cell surface	(83)
	Breast, cervical, liver cancer cells	Tumor cell adhesion to ECM, growth, EMT, metastasis, angiogenesis	Cell surface	(84, 231, 232, 233, 234, 235, 236)
	Platelets	Platelet activation	Cell surface	(85)
PSG	Placenta, sera during pregnancy	Cell invasion, immune tolerance, vascular remodeling	Cell surface	(90)
GM1	Neuroblastoma cells	Axon-like neuritogenesis	Cell surface	(92, 95, 96, 97, 235)
	T cells	Regulation of T cell activity	Cell surface	(93)
CD43	Dendritic cells	Regulation of autoimmune diseases	Cell surface	(101)
	Neutrophils	Cell migration and chemotaxis	Cell surface	(102)
CD45	Microglial cells	Inflammation	Cell surface	(103)
	B cells	Modulation of CD45 phosphatase activity	Cell surface	(109)
CD7	T cells	T cell viability	Cell surface	(106)
CD69	T cells	Th17 polarization	Cell surface	(108)
CD4/gp120	T cells/HIV	Cell infection	Cell surface	(111)
Laminin	Vascular SMCs	Cell adhesion, proliferation, spreading, migration	ECM	(72, 81)
Fibronectin	Vascular SMCs	Cell adhesion, migration	ECM	(72, 81)
Thrombospondin, osteopontin, vitronectin	Vascular SMCs	Cell adhesion	ECM	(72, 213, 214)
Heparan sulfate and chondroitin sulfate	Vascular SMCs	Cell adhesion to ECM proteins	ECM	(72, 213, 214)

**Table 2:** GAL-3-binding partners on different cell types

GAL-3-binding partner	Cell type/tissue	Function	Localization	References
α_v_ and β_3_ integrins	Endothelial cells	Angiogenesis	Cell surface	(112)
VEGFR-1/VEGFR-2	Endothelial cells	Angiogenesis	Cell surface	(43, 113)
JAG1 and DLL4 NOTCH ligands	Endothelial cells	Tumor angiogenesis, regulation of immune response	Cell surface	(115)
NOTCH-1	Ovarian cancer cells	Cancer progression, stemness	Cell surface	(117)
	Bone metastatic cancer cells	Osteoblast differentiation, bone remodeling, metastasis	Cell surface	(118)
CEA	Colon carcinoma cells	Cell adhesion, migration, metastasis	Cell surface	(126, 127)
TF/MUC1	Endothelial cells	Cancer cell adhesion to endothelium	Cell surface	(53, 128)
	Breast and colon cancer cells	Cancer cell adhesion to endothelium, transendothelial invasion, metastasis	Cell surface	(125, 221)
MUC1/MUC16	Corneal epithelium	Mucosal barrier maintenance	Cell surface	(131)
MUC16	Corneal keratinocytes	Binding of HSV-1 to mucins	Cell surface	(132)
CD146	Endothelial cells	Cell migration	Cell surface	(120, 121, 122)
CD13	Endothelial cells	Angiogenesis	Cell surface	(124)
LAMP-1/-2	Fibrosarcoma, melanoma and colon carcinoma cells	Invasion and metastasis	Cell surface	(125, 227)
EGFR	Breast cancer cells		Cell surface	(134, 135)
MERTK	Macrophages	Phagocytosis of apoptotic cells and cellular debris	Cell surface	(139)
TGF-βRII	Breast cancer cells	Receptor endocytosis	Cell surface	(134)
CD44	Fibroblasts Breast cancer cells	CD44 uptake, CLIC formation	Cell surface	(145)
GPVI	Platelets	Metastasis	Cell surface	(148)
TLR-4	Microglia cells	Inflammation	Cell surface	(149)
MICA	Bladder tumor cells	NK cell activation and tumor destruction	Cell surface	(150)
NKp30	NK cells	NK cell activation and degranulation	Cell surface	(151)
CTLA-4	T cells	Modulation of TCR signaling	Cell surface	(135)
LAG3	T cells	Regulation of tumor immune response	Cell surface	(154)
CD45	T cells	T-cell receptor signaling and T-cell survival	Cell surface	(104)
	Large B-cell lymphoma cells	Modulation of cell death	Cell surface	(155)
TfR (CD71)	T cells	Cell death	Cell surface	(104)
	HeLa cells	Endomembrane homeostasis	Lysosomal membrane	(156)
Laminin	Neutrophils	Cell adhesion and migration	ECM	(215)
Laminin, fibronectin and collagen-1	GE11 epithelial cells (mouse embryonic stem cells injected into blastocysts, chimeric embryos)	β_1_ integrin-mediated cell adhesion and migration	ECM	(216)
α_1_β_1_, α_5_β_1_, α_3_β_1_integrins	Corneal epithelial cells	Cell adhesion and migration	Cell surface	(217)
Collagen types I, IV, V, fibronectin, laminin-5			ECM

**Table 3:** GAL-4 -binding partners on different cell types

GAL-4-binding partner	Cell type/tissue	Function	References
CEA, GM1 ganglioside and glycosphingolipids	Colon cancer cells	Cell adhesion, migration, metastasis	(163)
TF	Colon cancer cells	Cancer cell adhesion to endothelium	(165)
TfR (CD71)	MDCK cells	Receptor apical transcytosis, trafficking to recycling endosomes and prevention of lysosomal targeting	(166)
CD3	T cells	Inflammation	(167)
CD14	Monocytes	Monocyte differentiation	(168)

**Table 4:** GAL-8-binding partners on different cell types

GAL-8-binding partner	Cell type/tissue	Function	Localization	References
*α*_3_, *α*_6_, *β*_1_ integrins	Non-small cell lung carcinoma cells	Cell adhesion	Cell surface	(170)
*β*_2_, *α*_4_ integrins	Jurkat T cells	Cell adhesion	Cell surface	(172)
*α*_M_ (CD11b) integrin	Neutrophils	Cell adhesion	Cell surface	(173)
*α*_1_, *α*_3_, *α*_5_ integrins	Jurkat T cells	Cell spreading	Cell surface	(174)
*α*_*L*_, *β*_*2*_ integrins	Peripheral blood mononuclear cells	Cell adhesion	Cell surface	(175)
Pro-MMP9	Neutrophils	Pro-MMP-9 processing by MMP3	Cell surface	(173)
GPIb	Platelets	Platelet activation	Cell surface	(176)
PDPN	Lymphatic endothelial cells	Lymphangiogenesis	Cell surface	(178, 179, 180)
VEGFR-3	Lymphatic endothelial cells	Lymphangiogenesis	Cell surface	(179)
ALCAM (CD166)	Vascular endothelial cells	Cell migration, angiogenesis	Cell surface	(181)
	Breast cancer cells	Cell adhesion, cell migration	Cell surface	(183)
CD45	Leukocytes	T cell proliferation	Cell surface	(185)
BCR	B cells	Antigen presentation B to helper T cells	Cell surface	(186)
CD44	Synoviocytes	Inflammation	Cell surface	(187)
Fibronectin	HeLa cells	Cell adhesion	ECM	(218)
	Lung carcinoma cells	Cell adhesion, metastasis	ECM	(219)

**Table 5:** GAL-9-binding partners on different cell types

GAL-9-binding partner	Cell type/tissue	Function	Localization	References
TIM-3	T cells	Th1 cell deletion	Cell surface	(190)
	Acute myeloid leukemia cells, leukemic stem cells	Immune surveillance, tumor progression	Cell surface	(191, 192, 193)
	Breast cancer cells	Tumor protection against cytotoxic immune attack	Cell surface	(194)
PD-1	T cells	Apoptosis modulation	Cell surface	(193)
VISTA	T cells	T cell granzyme B release	Cell surface	(195)
4-1BB	T cells, dendritic cells, NK cells	Inflammation	Cell surface	(196)
DR3	T cells	Immune cell homeostasis	Cell surface	(197)
CD44	T cells	TGF-βR signaling, inducible Treg differentiation	Cell surface	(198)
PDI	T cells	Th2 cell migration, HIV infection	Cell surface	(200)
CD45	B cells	B cell activation	Cell surface	(201, 202)
CD206	Melanoma-associated M2 macrophages	Angiogenesis	Cell surface	(203)
Dectin-1	Pancreatic adenocarcinoma-associated macrophages	Tumor progression, T cell activation	Cell surface	(204)
IgE	Mast cells	Asthmatic reaction, cutaneous anaphylaxis, regulation of mast cells activity	Cell surface	(205)
GLUT2	Pancreatic β cells	Receptor endocytosis	Cell surface	(208)
CD146	Blood–brain barrier (BBB) endothelial cells (BBBECs)	BBBEC adhesion to T cells	Cell surface	(209)
LAMP-1/-2	Hepatocarcinoma cells	Ubiquitination in response to lysosomal damage	Lysosomal membrane	(229)
	Gut epithelial cells	Lysosomal function	Lysosomal membrane	(230)

## Cell surface GAL-1-binding partners

### Neuropilins, vascular endothelial growth factor receptor 1 and vascular endothelial growth factor receptor 2

Neuropilins (NRPs) are highly conserved transmembrane glycoproteins. Two homologous NRP isoforms have been described -NRP1 and NRP2- encoded by distinct genes. NRPs have a large N-terminal extracellular domain, a short transmembrane domain, and a small cytoplasmic tail (32, 33). Both NRPs were originally discovered as neuronal adhesion molecules, acting as co-receptors for secreted class III semaphorins and participating in semaphorin-mediated axonal guidance. Semaphorin 3A (Sema3A) was described to prevent axonal regeneration through binding to the NRP1/PlexinA4 receptor complex after spinal cord injury (SCI) (34, 35). Interestingly, *in vivo* SCI model studies revealed the binding of dimeric GAL-1 to the NRP1/PlexinA4 receptor complex through a glycan-dependent mechanism. Through binding to the NRP1/PlexinA4 complex in injured neurons, GAL-1 treatment interrupted the Sema3A pathway and contributed to axonal regeneration and locomotor recovery after SCI (Table 1) (36).

In addition to their role in axonal regeneration, NRPs are also involved in vascular biology through binding to various growth factors, particularly vascular endothelial growth factor (VEGF), as well as transforming growth factor β (TGF-β), platelet-derived growth factors (PDGF) C and D, and c-Met (32, 33). Moreover, they also play important roles in immunity and tumorigenesis (32, 33). Remarkably, NRP1 was identified as a major receptor of GAL-1 on human umbilical vein endothelial cells (HUVECs) (37) (Table 1). Binding studies revealed high-affinity (dissociation constant (*Kd*): 109 ± 31 nM) and glycan-dependent binding of GAL-1 to recombinant NRP1, as demonstrated by surface plasmon resonance (SPR). This conclusion was further validated by flow cytometry and Western blot analysis in NRP1-silenced HUVECs (37).

NRP1 has also an important role in liver fibrosis and vascular changes involved in this process (38, 39). During liver fibrosis, activation of the PDGF/PDGFRβ axis is a key factor in the trans-differentiation of hepatic stellate cells (HSCs) into activated myofibroblasts. Activated HSCs proliferate and migrate into injured sites, secreting large amounts of ECM proteins which alter the normal hepatocyte parenchyma architecture and hepatic sinusoidal vascular structure (40). NRP1 is highly expressed on activated HSCs, where it regulates PDGF and TGF-β/SMAD signaling (38, 39). Notably, GAL-1 interacts with *N*-glycans present in NRP1 on LX-2 HSCs cells (41). Besides, GAL-1 induces PDGF- and TGF-β-like signals through the NRP1/PDGF receptor (PDGFR) and NRP1/TGF-β receptor (TGF-βR) complexes, thus modulating LX-2 cell signaling, activation and migration (41). In line with these findings, GAL-1 was also described to accelerate skin wound healing *in vivo* and to promote human gingival fibroblast proliferation, migration and activation through binding to NRP1 and triggering SMAD3/NADPH oxidase 4 (NOX4) signaling pathways (42). Thus, GAL-1/NRP1/PDGFR and GAL-1/NRP1/TGF-βR pathways critically regulate fibrosis, vascularization and myofibroblast biology.

As a co-receptor for VEGF, NRP1 forms complexes with VEGFR-1 and/or VEGFR-2 to enhance VEGF signaling, angiogenesis, cell migration, and tumorigenesis (32, 33). By means of proximity ligand assay (PLA), colocalization between VEGFR-1 or VEGFR-2 and early endosome antigen-1 (EEA1) was observed when endothelial cells were incubated with recombinant GAL-1. These findings suggested that GAL-1-glycan lattices may decrease VEGFR-1 and VEGFR-2 internalization, retaining these receptors on the cell surface (43). Interestingly, NRP1/VEGFR-1 complex formation and activation of the AKT/Rho A signaling pathway were required for GAL-1-induced HUVEC permeability (44). In addition, VEGFR-2 phosphorylation was detected following treatment of endothelial cells with recombinant GAL-1 (43, 45). Remarkably, GAL-1 signaling on endothelial cells was demonstrated to be VEGF-independent. Instead of altering VEGF signaling, GAL-1 directly stimulated the VEGFR-2 pathway by binding to complex non-sialylated *N*-glycans on this receptor. This conclusion was reached after performing co-immunoprecipitation experiments with HUVECs treated with GAL-1 in the presence of *N*-glycosidase F or following silencing of MGAT5 or core-2 β1-6-N-acetylglucosaminyltransferase 1 (C2GNT1), key glycosyltransferases responsible of generating GAL1 ligands. Furthermore, glycan-dependent interactions between GAL-1 and VEGFR-2 were confirmed by Förster Resonance Energy Transfer (FRET) analysis (45) (Table 1).

### CD146

CD146 (also known as melanoma cell adhesion molecule, MCAM, or cell surface glycoprotein MUC18) is a highly glycosylated junction adhesion molecule expressed on human vascular endothelial cells and many tumors (46, 47). Three variants of CD146 have been described in humans: two membrane-anchored forms (long and short) are encoded by the *CD146* gene and a soluble form of CD146 (sCD146) is generated by proteolytic cleavage of membrane forms. Both long and short isoforms are expressed on endothelial cells, whereas melanoma cells express mainly the long isoform (46). By ELISA and SPR, the sugar-dependent, direct and specific binding of CD146 to GAL-1 was demonstrated. Moreover, the interaction of this adhesion molecule with endogenous GAL-1 in HUVECs was observed by co-immunoprecipitation (48) (Table 1).

CD146 was recently found to be expressed in pre-adipocytes and mature adipocytes (49). This glycoprotein was described as an adipose receptor for angiopoietin-like protein 2 (ANGPTL2), which is involved in obesity-related chronic inflammation. Interestingly, co-immunoprecipitation experiments revealed that CD146 also interacted with GAL-1 in brown adipose tissue, an interaction that was confirmed by pull-down assays (49) (Table 1).

### Thomsen-Friedenreich (TF) glycotype and mucin 1 (MUC1)

The TF glycotype (Galβ1-3GalNAc) or CD176 is a tumor-associated glycoepitope (50). The presence of this antigen during early fetal life, its absence in post-fetal tissues, and its association with carcinomas suggest that the TF epitope is a stage-specific oncofetal carbohydrate epitope (50). It is expressed on both fetal epithelia and mesothelia. In normal adult human tissues, the TF disaccharide is expressed in limited amounts and restricted to a few immunologically privileged sites, such as the syncytiotrophoblast at the materno-fetal interface and extravillous trophoblast cells invading the decidua, and decorating transferrin from human amniotic fluid. In tumor cells, the transmembrane glycoprotein MUC1 is post-translationally modified, resulting in incomplete *O*-glycosylation and exposure of the TF epitope. Thus, TF antigen is expressed in most human carcinomas (50). Remarkably, the adhesion molecules and stem cell markers CD34 and CD44 also carry the unsubstituted TF antigen in certain types of cancer, including leukemia, colon, and breast carcinomas (51, 52).

GAL-1 was shown to cluster at contact sites between MDA-MB-435 human breast cancer cells and HUVECs, with a strong signal predominantly on cancer cells. As this heterotypic cell adhesion was inhibited in the presence of a TF antigen-specific P-30 peptide, the interaction between GAL-1 and TF antigen was first proposed (53) (Table 1). Then, exogenous biotinylated GAL-1 was described to bind TF in the syncytiotrophoblast and extravillous trophoblast layer from second-trimester human placenta and in BeWo chorioncarcinoma cells (54). Moreover, co-expression of TF epitope and MUC1, and binding of GAL-1 to TF antigen were also demonstrated in apical surfaces of human endometrial epithelial tissue in the early secretory phase and oocytes (55) as well as in endometrioid adenocarcinoma (56) (Table 1).

Interestingly, isothermal titration calorimetry (ITC) and mutagenesis studies showed that the TF disaccharide was not recognized by GAL-1 beyond millimolar affinity in solution and identified His^52^ as a key residue within the GAL-1 CRD that interferes with carbohydrate binding by steric hindrance (55, 57). Besides, mono- and trivalent TF presented on the MUC1 glycopeptide scaffold did not increase binding affinity for GAL-1 (57), highlighting the need of structural and mechanistic studies to definitely demonstrate the biological relevance of TF antigen recognition by GAL-1 within cell surfaces.

### Lysosome-associated membrane protein 1 and 2

Lysosome-associated membrane proteins (LAMPs) are a family of highly glycosylated transmembrane proteins. LAMP-1 and LAMP-2 (CD107a and CD107b) are ubiquitously expressed in human tissues and cell lines, mainly in the endosome-lysosomal membrane, but they are also found in the plasma membrane (58, 59, 60). LAMP-1 and LAMP-2 contain an *N*-glycosylated luminal (or extracellular) domain, a single-spanning transmembrane domain, and a short cytoplasmic tail which contain retrieval and targeting signatures (61, 62, 63). Given these structural features, LAMP-1 and LAMP-2 transport exogenous molecules from the plasma membrane to the lysosomes and generate a sugar coat or glycocalyx in the inner side of the lysosomal membrane protecting it from hydrolytic enzymes and degradation (59). These proteins are involved in a variety of cellular processes including phagocytosis, autophagy, lipid transport, aging, and cancer (60).

Surface expression of LAMP-1 and -2 depends on the cell type and physiological or pathological states. For instance, these proteins are expressed on the surface of activated monocytes and macrophages (64), in extracellular vesicles released from dendritic cells (65), and on tumor cell surfaces (60). Using affinity chromatography on GAL-1-agarose columns and immunoprecipitation, LAMP-1 and LAMP-2 were first identified as candidate GAL-1 receptors in Chinese Hamster Ovary (CHO), human A121 ovarian carcinoma and butyrate-differentiated KM12 colon carcinoma cell membrane extracts (66, 67, 68) (Table 1). Moreover, the interaction between LAMP-1/2 and GAL-1 was dependent on carbohydrate binding, and both LAMP-1 and LAMP-2 were detected on A121 and KM12 cell surfaces by immunofluorescence (67, 68). More recently, mass spectrometry analysis identified GAL-1 as a major protein present in secretory granules released by cytotoxic T cells, colocalized with granzyme B, perforin and LAMP-1/-2 (69) (Table 1).

### Carcinoembryonic antigen

Carcinoembryonic antigen (CEA) is a glycosylphosphatidylinositol (GPI)-anchored glycoprotein (70) derived from embryonic endodermal epithelium in the fetus. It usually vanishes from serum after birth; however, small quantities of CEA may remain in colon tissue. CEA is a non-specific serum biomarker that is elevated in various malignancies such as colorectal cancer, medullary thyroid cancer, breast cancer, mucinous ovarian cancer and others (71). CEA was reported as an endogenous receptor for GAL-1 in KM12 human colon carcinoma cell extracts, as demonstrated by affinity chromatography on immobilized GAL-1 followed by immunoprecipitation (68, 72). Furthermore, CEA isolated from colon carcinoma liver metastasis was found to bind GAL-1 in a carbohydrate-dependent manner (68).

### Integrins

Integrins are noncovalent heterodimers consisting of α and β subunits, which bind to the ECM. Each heterodimer combination has a selective affinity for ECM ligands, such as fibronectin, collagen, or laminin, and therefore the combination of integrin subunits expressed on individual cell types will determine its ability to bind particular ECM substrates (73). Integrins are ubiquitous, type I membrane glycoproteins with large extracellular domains, single transmembrane domains, and short intracellular tails (74). These proteins can be activated by “inside-out” or “outside-in” signaling. In the case of inside-out activation, signals are transmitted from non-integrin receptors, such as talin-1 to the β-subunit cytoplasmic tail, inducing conformational changes that turn integrins into an active state that binds extracellular ligands with higher affinity, thus promoting cell migration and ECM assembly and remodeling (75, 76, 77). In the case of outside-in activation mode, ligands bind to the external integrin domains and transmit signals into the cells (75). Consequently, integrins enable cells to adapt to variations in the extracellular environment, inducing changes in cell polarity, cytoskeletal structure, gene expression, cell survival, and proliferation. Moreover, deregulated integrin-mediated adhesion and signaling lead to the pathogenesis of many human diseases, including bleeding disorders, cardiovascular disorders, and cancer (78, 79).

Several integrins have been described as receptors for GAL-1 (Table 1). By affinity chromatography and Western blot experiments, Gu *et al.* (80) demonstrated that GAL-1 binds to α_7_β_1_ integrin on myoblasts in a lactose-dependent manner, and inhibits α_7_β_1_ integrin association with laminin, but not with fibronectin. Interestingly, recombinant glutathione S-transferase (GST)-GAL-1 fusion protein was observed to bind to α_1_β_1_ integrin by depletion of vascular smooth muscle cell (SMC) protein extracts with the corresponding antibody (81). By cross-linking ^125^I-labeled GAL-1 dimer to SMCs surface, GAL-1 was demonstrated to bind to a single β_1_ integrin molecule (82). Flow cytometry further demonstrated that GAL-1 enhanced β_1_ integrin activation on SMC surface (82). More recently, confocal microscopy, Western blot and co-immunoprecipitation assays revealed that GAL-1 binds to β_1_ integrin, but not to α_1_ or α_5_ subunits, at the plasma membrane of human trophoblasts (83). Similar results were obtained in MDA-MB-231 and Hs578T human breast cancer cell lines (84). Structural modeling studies using crystal structures of human α_5_β_1_ integrin (Protein Data Bank (PDB) code: 4WJK) and human homodimeric GAL-1 (PDB: 1GZW) supported the potential interaction between β_1_ integrin *N*-linked glycans and dimeric GAL-1 CRDs (83). By GAL-1-affinity chromatography and mass spectrometry studies, α_IIb_β_3_ integrin was also demonstrated to interact with GAL-1 in human platelets (85) (Table 1).

### Pregnancy-specific β-glycoprotein 1

Pregnancy-specific β-glycoprotein 1 (PSG1) is secreted from trophoblasts during pregnancy and exerts immunomodulatory and pro-angiogenic functions (86). It is comprised of four immunoglobulin-like domains (N, A1, A2, and B2) (87), which interact with distinct ligands. For example, PSG1 binds to α_IIb_β_3_ integrin and inhibits its interaction with fibrinogen (88). Furthermore, PSG1 modulates the adhesion and migration of extravillous trophoblasts through binding to α_5_β_1_ integrin (89). Complete glycomics and glycoproteomics analysis on native PSG1 from sera of pregnant women revealed the presence of multianntennary and multiple LacNAc elongated moieties with mainly α2-3-linked sialic acid terminals (90), suggesting that PSG1 could be a potential galectin ligand. Notably, ELISA and SPR experiments confirmed the interaction between human GAL-1 and PSG1, in a lactose-dependent manner (90) (Table 1). Thus, GAL-1, PSG1, and integrins might cooperate to modulate cell invasion, immune tolerance, and vascular remodeling during pregnancy.

### GM1 ganglioside

Gangliosides are cell-type-specific sialic acid-containing glycosphingolipids abundant in the mammalian brain. Ganglioside GM1 is a sialotetraosyl ceramide consisting of a branched pentasaccharide made up of one sialyl residue, two galactose residues, one *N*-acetylgalactosamine, and a glucose residue at the reducing end attached to *N*-stearoylsphingosine (91). GAL-1 has been demonstrated to bind GM1 at the surface of human neuroblastoma cells and murine T cells (92, 93) (Table 1). GAL-1 binding to GM1 was confirmed using biotinylated GAL-1, neuraminidase treatment, and high-performance thin-layer chromatography (HPTLC) (93, 94). Interestingly, GAL-1 cross-linking of β_1_ integrin-associated GM1 triggered Ca^2+^ influx *via* transient receptor potential canonical subgroup 5 (TRPC5) channels (93, 94). Regarding structural analysis, both molecular modeling and nuclear magnetic resonance (NMR) studies showed that the GM1 galactose residues are potential binding sites for GAL-1 and that this saccharide moiety accommodates in the vicinity of Trp^68^ within GAL-1 CRD (95). Moreover, X-Ray scattering data of 1,2-dihexadecanoyl-sn-glycero-3-phosphoethanolamine (DPPE)-GM1 monolayers at an air/buffer interface revealed that GAL-1 is oriented with its long axis in the surface plane, ideal for *cis*-cross-linking (96) (Table 1).

Concerning the biological consequences of this interaction, GAL-1 was shown to exert growth control through GM1 binding on human SK-N-MC neuroblastoma cells and activated effector T cells (92, 93) (Table 1). Besides, by cross-linking GM1 and its associated glycoprotein α_5_β_1_-integrin, GAL-1 elicited axon-like neuritogenesis and triggered regulatory T (Treg) cell activity (93, 94). Although chimeric GAL-3 shares binding parameters with proto-typical GAL-1 for SK-N-MC neuroblastoma cells, it failed to influence cell growth by itself, but interfered with GAL-1 effect, probably through competitive inhibition (92, 97). In contrast, the tandem-repeat-type GAL-4 was neither a growth modulator nor a competitive inhibitor for GAL-1 (98). Therefore, different CRD topological presentations of diverse galectin members might affect GM1 cross-linking and cell responses. Further, GAL-1 interaction with GM1 may be relevant in axon regeneration and suppression of autoimmune diseases (92, 93).

### CD44

CD44 is a non-kinase cell surface transmembrane glycoprotein recognized as the main receptor for hyaluronan, an extracellular glycosaminoglycan, which is the major ECM component. CD44 is a member of the cell adhesion molecules (CAMs) family that plays essential roles in cellular communication and adhesion between cells and the ECM. The *CD44* gene undergoes alternative splicing, resulting in the standard (CD44s) and variant (CD44v) isoforms. The interaction of such isoforms with specific ligands, particularly hyaluronic acid, osteopontin, and matrix metalloproteinases (MMPs), triggers different cell signaling events (99). Recently, a proximity tagging strategy was used to identify GAL-1-interacting glycoproteins in mouse myoblasts (100). In this approach, a fusion protein of GAL-1 and an engineered heme-containing ascorbate peroxidase enzyme was generated and applied to cells to covalently tag proximally located interactors with affinity labels for capture enrichment, and identification by quantitative mass spectrometry-based proteomics. Although many *N*-linked and *O*-linked glycoproteins were identified as GAL-1-binding partners, only the interaction with CD44 was validated by immunoprecipitation, and the requirement of CD44 *N*-linked glycans for this association was confirmed (100).

### CD43

Regarding innate immune responses, GAL-1 has been shown to bind to CD43 sialomucin on dendritic cells in a glycan-dependent fashion, interfering with terminal differentiation and inducing a regulatory profile. GAL-1-conditioned dendritic cells expressed high levels of the immunosuppressive cytokine IL-27 and favored the induction of IL-10-producing type 1 regulatory T (Tr1) cells, which contributed to suppressing autoimmune neuroinflammation (101). Mechanistically, GAL-1 induced CD43 membrane segregation and promoted phosphorylation of the STAT3 transcription factor. In addition, in human neutrophils, CD43-GAL-1 interaction increased migration and chemotaxis (102).

### CD45

In microglial cells, GAL-1 has been demonstrated to interact with the CD45 phosphatase in an *O*-glycan-dependent manner. These specialized cells of the central nervous system are activated during inflammation and can cause neurodegeneration. GAL-1-*O*-glycan interactions attenuate microglial activation through increased CD45 phosphatase activity, leading to decreased phosphorylation of p38-, CREB- and NF-κB-dependent pathways and suppression of inflammation-driven neurodegeneration (103).

Regarding its roles in adaptive immunity, GAL-1 can modulate T and B cell activation, differentiation, and apoptosis (104, 105, 106, 107, 108). Different receptors have been found to bind this glycan-binding protein on T cells. Like myeloid cells, CD45 expressed on T cells interacts with GAL-1, leading to membrane segregation and acceleration of T cell apoptosis (104). Interestingly, GAL-1-induced T-cell death required the presence of core two *O*-glycans on CD45 (105).

GAL-1 binding to CD45 was further supported by co-immunoprecipitation experiments in Raji B cells, and CRISPR-Cas9-mediated GAL-1 knockout promoted a striking reduction of CD45 phosphatase activity in these cells (109). Remarkably, T helper signals were demonstrated to increase CD45 phosphatase activity on these cells by amplifying GAL-1 binding to the cell surface (109). In this regard, differential glycosylation of Th1 and Th17 cells has been shown to modulate the survival and function of these cells by enabling selective exposure of GAL-1-specific glycoepitopes through mechanisms involving CD45 segregation (110).

### CD7, CD69, and CD4/gp120

Other glycosylated receptors were also found to bind GAL-1 on T cells, including CD7 which functions as a potential GAL-1 ligand implicated in T-cell death (106) and CD69, a known T cell activation marker, which inhibits Th17 polarization and JAK3/STAT5 signaling when engaged by GAL-1 (108). Furthermore, in the context of human HIV-1 infection, GAL-1 mediates the interactions of the CD4 receptor and the gp120 envelope protein, thus facilitating virus adhesion and infection. Spacial orientation of complex *N*-glycans on gp120 permitted GAL-1 binding while impairing interaction with GAL-3 (111).

## Cell surface GAL-3-binding partners

### Integrins

Several glycoproteins on the surface of endothelial cells have been implicated in GAL-3-mediated angiogenesis (Table 2). As an example, integrins were identified as receptors for GAL-3 on these cells. Affinity chromatography on GAL-3-immobilized columns followed by mass spectrometry analysis revealed that α_v_ and β_3_ integrin subunits are important GAL-3-binding partners on HUVECs. Notably, knocking-down MGAT5 in HUVECs eliminated the pro-angiogenic activity of GAL-3, highlighting the relevance of β1,6GlcNAc-branched *N*-glycans on α_v_β_3_ integrin in GAL-3 binding to endothelial cells (112) (Table 2).

### VEGFR-1 and VEGFR-2

Similar to GAL-1, GAL-3 was also demonstrated to activate and directly interact with VEGFR-2 on HUVECs plasma membrane in an MGAT5-dependent manner. Using both MGAT5 and GAL-3 knockdown cells, and performing confocal microscopy and biotin-based endocytosis assays, GAL-3 was demonstrated to reduce VEGFR-2 internalization, thus retaining this receptor on the plasma membrane upon VEGF-A stimulation through glycosylation-dependent mechanisms (113). Moreover, the addition of GAL-3 to endothelial cells induced VEGFR-1 and VEGFR-2 phosphorylation while promoting a decrease in both VEGFR-1 and VEGFR-2 endocytic pools (43) (Table 2).

### NOTCH-1 receptor, Delta-like canonical NOTCH ligand 4, and Jagged-1

A widely accepted model of angiogenesis states that VEGF/VEGFR-2 signaling increases the levels of Delta-like canonical NOTCH ligand 4 (DLL4) on endothelial tip cells, which then signals *via* the NOTCH receptor on adjacent endothelial cells, favoring the induction of stalk cells and blocking VEGFR-2 expression and excessive sprouting (114). Inhibiting DLL4/NOTCH has been shown to reduce tumor growth by promoting the proliferation of immature vessels, leading to deficient vascular perfusion. While DLL4 has an anti-angiogenic function, Jagged-1 (JAG1) expression on endothelial cells has been shown to promote angiogenesis by antagonizing DLL4/NOTCH signaling and promoting vascular maturation. Therefore, DLL4 and JAG1 have opposing roles in angiogenesis (114). NOTCH ligands are cell surface glycoproteins containing terminal β-galactose and GAL-3 CRD was demonstrated to interact with both recombinant human JAG1 and DLL4 in ELISA assays, being these interactions blocked by lactose. Moreover, direct GAL-3 binding to JAG1 and DLL4 was confirmed by SPR assay (*Kd* 8.913 ± 0.485 μM for JAG1, *Kd* 8.523 ± 0.335 μM for DLL4). Interestingly, although GAL-3 was capable of binding to both ligands, it selectively increased the JAG1 half-life and its accumulation at the HUVEC surface. Besides, tumor-secreted GAL-3 increased JAG1/NOTCH signaling in endothelial cells and this activation was independent of VEGF/VEGFR-2 signaling (115, 116).

When NOTCH ligands, including JAG1, JAG2, DLL1, DLL3, and DLL4, interact with NOTCH transmembrane receptors on adjacent cells, the cleavage of NOTCH receptor to release NOTCH-1 intracellular domain (NICD1) is induced by proteases. Then, NICD1 translocates to the nucleus and binds to DNA-binding proteins to assemble a transcription complex that activates downstream target genes (114). Remarkably, when GAL-3 was silenced or overexpressed in ovarian cancer cells, there was a decrease or increase in NICD1 cleavage, respectively, as well as changes in the expression of the NOTCH target genes *Hes1* and *Hey1*. Additionally, by co-immunoprecipitation, GAL-3 CRD was demonstrated to interact with NOTCH-1 and NICD1, and GAL-3 overexpression increased NICD1 nuclear translocation (117). Furthermore, soluble GAL-3 regulated bone remodeling by binding to NOTCH-1 and activating NOTCH signaling in a sugar-dependent manner (118). However, there is also evidence suggesting that GAL-3 plays a role in reducing the activation of NOTCH signaling in the immune system. Regulatory and effector T cells isolated from GAL-3-deficient mice showed higher levels of NOTCH-1 expression and the NOTCH target gene *Hes-1*. Lack of GAL-3 in these mice also increased JAG1/NOTCH activation in bone marrow-derived dendritic cells, which in turn led to disruption of T helper cell polarization (119). Thus, depending on the cell type and cellular context, and through binding to NOTCH-1 and NOTCH ligands, GAL-3 may activate or inhibit NOTCH signaling to modulate tumor angiogenesis and immune responses.

### CD146

Through affinity purification, mass spectrometry, and co-immunoprecipitation assays, CD146 was identified as a GAL-3-binding partner in vascular endothelial cells. Notably, the binding of GAL-3 to *N*-linked glycans induced CD146 dimerization and subsequent activation of AKT signaling (120) (Table 2). Pull-down assays, gel filtration, bio-layer interferometry, and mass spectrometry approaches with CD146 recombinant ectodomain and GAL-3 confirmed their direct interaction with a *Kd* of approximately 1.1 μM, and critical involvement of poly-LacNAc units on domain 5 (D5) of CD146 ectodomain (121). Moreover, the first structural study of GAL-3 binding to a cell surface glycoprotein receptor was carried out by NMR ^15^N-Heteronuclear Single Quantum Coherence (HSQC) spectroscopy and biolayer interferometry (122). Binding between ^15^N-labeled GAL-3 (full-length and truncated CRD) and the extracellular portion of CD146 (domains D1–D5, residues 1–559) or its D5-truncated variant (D1–D4, residues 1–424) contributed to a better understanding of the key role of CD146 ectodomain D5 in this association. Although the canonical carbohydrate-binding β-sheet S-face (β-strands 1, 10, 3, 4, 5, 6) of the GAL-3 β-sandwich was involved in the interaction, the opposing GAL-3 F-face β-sheet (β-strands 11, 2, 7, 8, 9) was demonstrated to be critical for CD146 D5 binding (122).

### CD13 (Aminopeptidase N)

CD13 is a heavily glycosylated type II zinc-binding membrane metallopeptidase (123). Using phage display biopanning, GAL-3 was identified as a major binding partner for CD13 in HUVECs. Further, SPR analysis and colocalization studies demonstrated the carbohydrate-dependent interaction between GAL-3 and CD13 with a *Kd* of 0.349 μM. In addition, results obtained from HUVEC invasion and tube formation assays revealed the functional relevance of CD13 as an important receptor for GAL-3 in the endothelium (124) (Table 2).

### LAMP-1, LAMP-2, and CEA

LAMP-1 and LAMP-2 have also been identified as potential GAL-3 ligands (Table 2). By flow cytometry, GAL-3 was shown to bind LAMP-1 and LAMP-2 at the surface of A2058 melanoma, HT1080 fibrosarcoma, and CaCo-2 colon carcinoma cells (125). Likewise, CEA was also detected as a GAL-3-binding partner in human colon carcinoma cells (126). This antigen was demonstrated to interact with GAL-3 at the cell surface of colorectal carcinoma cells by PLA assays and to colocalize with GAL-3 in colorectal carcinoma patient tissues (127).

### TF, MUC1, and MUC16

The tumor-associated carbohydrate TF is also recognized by GAL-3. By interacting with TF, GAL-3 induced breast cancer cell adhesion to the endothelium (53, 128). Furthermore, in co-immunoprecipitation experiments, the TF epitope on the MUC1 glycoprotein was found as a natural ligand of endogenous GAL-3 in human HT29 colon cancer cells (125). Remarkably, ITC assays showed that TF antigen affinity for GAL-3 CRD was two orders of magnitude higher than that for GAL-1 (*Kd* 47 μM *versus* 4 mM), demonstrating the different binding capacities of galectins for individual ligands (129) (Table 2). Mechanistically, crystal structures of GAL-3 CRD-TF antigen complex as well as mutagenesis experiments and detailed structural analyses comparing GAL-1 and GAL-3 CRDs, identified a penta-residue motif (^51^AHGDA^55^) at the loop L4 of GAL-1 (g1-L4) connecting GAL-1 β-strands 4 and 5, whereas the corresponding one in GAL-3 (g3-L4) constitutes a tetra-residue motif (^165^ENNR^168^). Thus, the presence of His^52^ residue in GAL-1 loop g1-L4 causes a narrower cavity for TF antigen binding (129). These results suggested that in a cellular context, the TF antigen might be recognized *via* modification of the penta-residue motif to trigger a conformational change in the g1-L4 loop similar to that observed in g3-L4 for GAL-3 (129). More recently, the presentation of TF antigen by MUC1 was demonstrated to enhance GAL-3 recognition by 10 folds, highlighting the relevance of the cellular context in the interaction between GAL-3 and TF antigen (130).

MUC1 and another highly *O*-glycosylated transmembrane mucin, MUC16, were identified as receptors for GAL-3 at the apical membrane of the corneal epithelium (Table 2). Affinity chromatography, immunofluorescence microscopy, cell surface biotinylation and colocalization studies, together with down-regulation of C1GALT1, the galactosyltransferase required for the synthesis of core one *O*-glycans (precursor for many extended mucin-type *O*-glycan structures), demonstrated that GAL-3 binds to *O*-glycosylated MUC1 and MUC16 on the apical surface of epithelial cells (131). In line with these findings, GAL-3 knockdown strategies in a 3D cell culture system of human corneal keratinocytes confirmed the interaction between GAL-3 and MUC16 and revealed its critical role in preventing binding of herpes simplex virus type 1 (HSV-1) to mucins (132).

### Epidermal growth factor receptor (EGFR)

Epidermal growth factor receptor (EGFR) is a member of the ERBB family of receptor tyrosine kinases (RTKs). The EGFR-mediated pathways are important in diverse cellular processes, including cell proliferation, differentiation, survival, and tumorigenesis (133). Association of GAL-3 with EGFR has been demonstrated by immunoprecipitation following cross-linking of cell surface mammary tumor cells. Accordingly, reduced GAL-3 binding to complex *N*-glycans on EGFR was verified in MGAT5^−/−^ cells (134, 135). While lactose pretreatment of wild-type cells blocked GAL-3 and EGFR association, *N*-acetylglucosamine restored surface levels of EGFR bound to GAL-3 in *MGAT5*^*−/−*^ cells (134, 135), demonstrating the importance of complex branched *N*-glycans in GAL-3 binding to EGFR. Importantly, increased colocalization of EGFR with early endosomal marker EEA-1 was observed in *MGAT5*^*−/−*^ cells, suggesting that GAL-3-EGFR cross-linking at the cell surface may restrict receptor internalization and maintain signaling activation (134). Remarkably, affinity chromatography, mass spectrometry and SPR studies unveiled the ability of GAL-3 to bind to the extracellular glycosylated domain of MUC1, serving as a link between EGFR and this mucin (136) (Table 2).

### MER tyrosine kinase (MERTK)

The TAM receptor protein tyrosine kinases -TYRO3, AXL, and MER- share significant structural features but may have different post-translational modifications, including glycosylation, phosphorylation, and ubiquitination. TAMs also share two homologous ligands, the vitamin K-dependent proteins GAS6 and S1 (137). In adult tissues, TAMs are widely distributed and up-regulated in numerous cancers. They have important roles in cell growth, proliferation and differentiation of normal cells, and contribute to resistance to immune or radiation therapy in cancer cells (138).

In addition to canonical TAM ligands, non-canonical ligands have been described (138). In this regard, the dual functional cloning approach, in which phagocytosis-based functional selection was combined with receptor-based affinity selection, revealed that GAL-3 may serve as a novel non-canonical ligand capable of binding to MERTK (139) (Table 2). Functional activity, co-immunoprecipitation, receptor activation, and functional blockade experiments confirmed this finding. GAL-3 activation of MERTK was shown to stimulate phagocytosis of apoptotic cells and cellular debris by macrophages and retinal pigment epithelial cells (139). More recently, GAL-3 was also shown to stimulate TYRO3 phosphorylation and activation, triggering ERK and AKT signal transduction pathways, thereby promoting tumor cell proliferation, survival, and migration in human head and neck squamous cell carcinoma and bladder cancer cell lines (140). Whether the GAL-3 CRD interacts with TYRO3 glycans remains to be explored.

### TGF-βR type II (TGF-βRII)

The TGF-β family ligands are multifunctional cytokines that signal *via* heterotetrameric complexes of type I and type II kinase receptors (141). Mammary tumor cells derived from *Mgat5*^−/−^ mice displayed reduced GAL-3 binding to TGF-βRII, demonstrating that this lectin can form multivalent complexes with TGF-βRII on the cell surface, thus preventing receptor endocytosis through *N*-glycan-dependent mechanisms (134).

### CD44

From a mechanistic perspective, endocytic events can be classified into two types: clathrin-dependent and clathrin-independent processes (142). The hyaluronan receptor CD44 is an example of a cargo that can be effectively internalized in the absence of clathrin (143). This receptor was assigned to early internalization structures with a particular morphology called clathrin-independent carriers (CLICs) (144). CLICs originate directly from the plasma membrane, mature into glycosylphosphatidylinositol-enriched early endosomal compartments, and merge with early endosomes (142). Research involving super-resolution and reconstitution techniques revealed that GAL-3 promotes clustering and membrane bending and thus, contributes to the biogenesis of CLICs in fibroblasts and breast tumor cells in a glycosphingolipid-dependent manner. In fact, GAL-3 interacts with *N*-glycosylated branches present in CD44 and β_1_ integrin cargo proteins. Moreover, GAL-3 and glycosphingolipids were essential for the uptake of CD44 and β_1_ integrin as well as for CLIC formation (145).

### Glycoprotein VI

Platelet membrane receptors are critical mediators of platelet interaction with tumor cells, thus influencing cancer cell survival and dissemination (146). Glycoprotein VI (GPVI) is a receptor for collagen, laminin, and fibrin, which regulates multiple platelet functions, such as adhesion, aggregation, and procoagulant activity (147). GPVI is a type I transmembrane glycoprotein belonging to the immunoglobulin superfamily that contains an extracellular chain with two collagen-binding Ig-C2-like domains, a transmembrane region, and a cytoplasmatic tail (147). Recently, using bioinformatics tools and the available protein structure crystalography information, GPVI was identified as a receptor for GAL-3 with a binding affinity (ΔG_bind_) of −5.91 kcal/mol and a *Kd* of 44.89 mM, estimated using the umbrella sampling technique, which were similar to those found for GPVI and collagen (148). By using GPVI-conjugated fluorescent microspheres and static adhesion assays in CRISPR/Cas9-GAL-3-deficient colon (MC38) and breast cancer (AT-3, 4T1, and E0771) cells, the collagen-like domain was found to be essential in GAL-3-GPVI interactions (148) (Table 2). Furthermore, experimental and spontaneous models of tumor metastasis using GAL-3-deficient tumor cells and *GPVI*^−/−^ mice confirmed that platelets promote metastasis of colon and breast cancer cells through association of platelet GPVI and GAL-3 (148).

### Toll-like receptor 4, major histocompatibility complex class I-related chain A, and NKp30

GAL-3 plays key roles in immune modulation, displaying context-dependent pro- or anti-inflammatory activities (8). GAL-3 has been shown to bind to Toll-like receptor 4 (TLR4) on microglia cells and promote central nervous system inflammation (149). Moreover, in NK cells, GAL-3 interfered with both NKG2D- and NKp30-mediated activation in a glycan-dependent manner. In C2GNT1-expressing tumor cells, GAL-3 was demonstrated to bind to core two *O*-glycans present in major histocompatibility complex class I-related chain A (MICA), which impeded ligand binding to NKG2D and subsequent NK cell activation and tumor destruction (150). On the other hand, tumor-derived GAL-3 has been shown to directly bind to NKp30, thus impairing NK cell activation and degranulation (151).

### CD4, CD8, cytotoxic T lymphocyte-associated antigen-4 (CTLA-4) and lymphocyte activation gene-3 (LAG3)

Within the T cell compartment, GAL-3 has been shown to prevent T cell receptor (TCR)-mediated activation and association with both CD4 and CD8 co-receptors (152). Moreover, GAL-3 attenuated TCR signaling by antagonizing ALIX, a cytoplasmic protein that controls endocytosis and exosome function (153). Interestingly, with particular relevance in cancer immunotherapy, GAL-3 was found to interact with both CTLA-4 and LAG3, two clinically-relevant immune checkpoint molecules. In the first case, GAL-3 associated to complex branched *N*-glycans, increasing CTLA-4 retention on the cell surface and downregulating TCR signal strength (135). On the other hand, GAL-3 has been found to promote CD8 T cell dysfunction by engaging LAG-3 (154).

### CD45

Additional GAL-3-binding partners on T cells have been identified by loading cell surface proteins onto a GAL-3-affinity column, eluting them with lactose, and processing them by mass spectrometry and immunoprecipitation (104). Retained glycoproteins included CD45, CD29 (or β_1_ integrin) and CD43. Notably, CD45, but not CD29 nor CD43, were implicated in the regulation of T-cell death programs triggered by GAL-3. Likewise, in a setting of large B-cell lymphoma, GAL-3 bound to CD45 and modulated its phosphatase activity, which contributed to apoptosis resistance (155).

### Transferrin receptor (TfR or CD71)

TfR was also described as a GAL-3-binding partner on T cells. GAL-3 induced clustering of TfR (CD71), but not CD45, on the T cell surface. While TfR is uniformly distributed on the T cell surface, GAL-3 binding led to TfR localization into distinct cell surface clusters or patches, particularly on dying cells, suggesting the potential roles of TfR (CD71)-GAL-3 interactions in modulating T cell viability (104).

Glycosylated TfR was also found to be recognized by GAL-3 at the lysosomal membrane to signal damage (156). When organellar and plasma membrane are damaged, the endosomal sorting complexes required for transport (ESCRT) and autophagy machinery contribute to restoring homeostasis (157, 158, 159). Remarkably, GAL-3 has been described to be involved in autophagy during lysosomal damage (160). Similar to T cells, the treatment of HeLa cells with a lysosomal membrane-damaging agent revealed the importance of ALIX as a cytoplasmic binding-protein of GAL-3 (156). ALIX is a specific ESCRT component recruited directly to membrane damage sites on lysosomes (161). Structurally, the N-terminal oligomerization domain of GAL-3 was demonstrated to interact with a proline-rich region of ALIX (162). Super-resolution microscopy (direct stochastic optical reconstruction microscopy) and high-content microscopy quantification confirmed that GAL-3 and ALIX interact, colocalize, and co-recruit to damaged lysosomal organelles. Using Lec3.2.8.1 mutant CHO cells lacking *N*-glycans it was demonstrated that damage-exposed lysosomal glycosylation was critical for GAL-3 binding and recruitment of ALIX to damaged lysosomes. Dynamic proteomic analysis by proximity biotinylation confirmed the importance of TfR as a receptor for GAL-3 within the lysosomal membrane and its critical role in signaling damage and engaging ALIX. The association between GAL-3 and ALIX was completely abolished after silencing TfR, suggesting a key role for this receptor in GAL-3-ALIX interaction. GAL-3 also facilitated the transition to ESCRT-III complex formation to sites of lysosomal damage. Thus, during lysosomal damage and through the association with TfR and ALIX, GAL-3 coordinated both ESCRT-dependent repair and autophagy-dependent removal systems to restore endomembrane homeostasis (156). In summary, glycosylated TfR (CD71) serves as a major binding partner of GAL-3 both at the plasma and lysosomal membranes.

## Cell surface GAL-4-binding partners

### CEA, GM1 ganglioside, and glycosphingolipids

The tandem repeat-type GAL-4 has been shown to bind CEA in human colon adenocarcinoma LS174T cells. This interaction showed a *Kd* value of 2 × 10^−8^ M as demonstrated by SPR. Moreover, this lectin also interacts with GM1 ganglioside and glycosphingolipids carrying 3-*O*-sulfated galactose residues, such as SB1a, SB2, SM3, SM4s, and SM2a ceramides (163). GAL-4 colocalized in patches with SB1a, GM1, and CEA on the cell surface of human colon adenocarcinoma CCK-81 and LS174T cells, and this localization differed from caveolin staining. Thus, SB1a and CEA serve as functional ligands of GAL-4 in human colon adenocarcinoma cells (163). Notably, although both N-GAL-4 and C-GAL-4 domains bound to glycosphingolipids, the affinity of GST-C-domain toward 3-*O*-sulfated glycosphingolipids SB1a and SM3 was higher than that of GST-N-domain. This GAL-4 feature diverges from that of GAL-8, where only the N-terminal domain has an affinity for glycosphingolipids (164).

### TF and MUC1

Like other members of the family, TF antigen in MUC1 was also demonstrated to mediate GAL-4-driven adhesion of cancer cells to the endothelium. Results obtained by ELISA demonstrated that GAL-4 recognized unsubstituted and terminal TF disaccharide. Removal of TF antigen from human HT29-5F7 colon cancer cell surface by *O*-glyconase treatment reduced Gal-4-driven tumor cell adhesion to human microvascular endothelial cells (HMVEC). These findings were further confirmed by silencing MUC1 expression in HT29-5F7 cells, which significantly diminished the effect of GAL-4 on heterotypic cell adhesion (165) (Table 3).

### TfR (CD71)

GAL-4 was also demonstrated to bind to TfR and to mediate basolateral to apical epithelial transcytosis (166). AP-1B is a clathrin-interacting adaptor that is expressed by most columnar epithelia and regulates the sorting of basolateral proteins. GAL-4 effects on transcytosis were studied using an AP-1B knockdown approach in Madin-Darby canine kidney (MDCK) cells, as well as retinal pigment epithelial (RPE) and kidney proximal tubule (KTP) cell lines. Apical transcytosis of TfR in AP-1B-deficient epithelia required an apical sorting event, mediated by specific signals. Interestingly, GAL-4 silencing inhibited TfR transcytosis to apical recycling endosomes and the apical plasma membrane and promoted TfR lysosomal targeting and subsequent degradation. Functional ablation of *N*-glycans on N^727^ residue in TfR also inhibited its apical transcytosis and increased its lysosomal degradation. Moreover, the mutant N727A-TfR-GFP remained mostly basolateral in AP-1B knockdown-MDCK cells, indicating that this TfR mutant was not transcytosed to the apical plasma membrane on these cells. In particular, GAL-4 knockdown (but not GAL-3 knockdown) reduced the colocalization of basolaterally internalized fluorescent transferrin (CF594–Tf) with Rab11a, a recycling endosome marker, but increased the colocalization of basolaterally internalized CF594–Tf with the lysosomal marker LAMP-1, as compared to controls in AP-1B-knockdown/TfR MDCK cells. Taken together, these results indicate that GAL-4 mediates TfR apical transcytosis in AP-1B knockdown MDCK cells, by promoting its trafficking to recycling endosomes and preventing its lysosomal targeting (166).

### CD3

Similar to GAL-1, GAL-4 binds to CD3, a surface protein associated with the TCR and implicated in antigen recognition and signaling (167, 168). At the functional level, exposure to GAL-4 resulted in potent inhibition of peripheral blood T cell cycling. When peripheral blood mononuclear cells were activated with anti-CD3 antibodies and incubated with recombinant GAL-4, expression of the co-stimulatory molecules CD80 and CD86 was significantly inhibited. Besides, GAL-4 potently reduced the secretion of pro-inflammatory cytokines including TNF-α, IL-8, IL-10, and IL-17 in peripheral blood T cells. In GAL-4-treated mice, peripheral blood mononuclear cells also showed reduced secretion of TNF-α, but not IFN-γ. With regard to anti-inflammatory cytokines, GAL-4 increased IL-10 secretion compared to controls. Likewise, in a model of mucosal cell cytokine secretion in colon culture systems, GAL-4 treatment also reduced TNF-α secretion and increased IL-10 production, while IFN-γ secretion did not change. In summary, GAL-4 potently binds to activated T cells *via* CD3, inducing potent inhibition of cell cycle progression and favoring induction of antigen-induced cell death (167).

### CD14

CD14 has also been postulated as a GAL-4 binding partner (169). CD14 is a surface protein preferentially expressed on monocytes and macrophages, which acts as a co-receptor for either TLR4 or MD-2 upon sensing of bacterial LPS. Results showed that GAL-4 strongly interacted with CD14 but not with TLR-2, -4, or -6 on monocytes. Anti-CD14 blocking antibodies diminished the granularity and cytokine production in GAL-4-treated monocytes. The activation of the CD14-TLR4 signaling pathway recruits Toll/IL-1 receptor (TIR) domain-containing adaptors and initiates downstream inflammatory cascades such as cytokine production. One of the critical TIR domain adaptor proteins, myeloid differentiation primary response 88 (MyD88), activates NF-κB and MAPK system comprised of the ERKs, JNK, and the p38 MAPK. In fact, GAL-4 significantly increased the expression of macrophage marker Ly6C in peripheral blood mononuclear cells from wild-type mice, but not from MyD88-deficient mice. GAL-4 treatment also increased p38, JNK, and ERK phosphorylation. Moreover, expression of CCR1 and CCR5 were up-regulated in GAL-4-treated monocytes, whereas CCR2 and CXCR4 levels were decreased. Significantly higher levels of IL-6, IL-10, and TNF-α were detected in the supernatant of GAL-4-treated monocytes. However, cytokine levels were reduced as compared to LPS-treated controls, and unlike LPS treatment, GAL-4 did not enhance IL-1β and IL-12 production. Increased IL-6 levels in CD14^+^ cells were observed rapidly after GAL-4 treatment. Notably, GAL-4 treatment significantly increased macrophage cell markers, particularly CD64, but did not induce any change in dendritic cell markers. Thus, CD14 emerges as a strong candidate receptor for GAL-4, which triggers TLR4-mediated MAPK signaling cascades. In summary, GAL-4 binding to CD14 promotes differentiation of monocytes into unique macrophage-like cells (169). Further studies are warranted to elucidate the mechanisms underlying the differential pro- or anti-inflammatory activities of this tandem-repeat-like galectin.

## Cell surface GAL-8-binding partners

### Integrins and glycoprotein (GP) Ib (CD42b)

Different integrins have been reported as putative GAL-8 ligands, depending mainly on the integrin repertoire expressed by each cell type (170, 171, 172) (Table 4). In neutrophils, affinity purification of galectin-interacting proteins followed by N-terminal amino acid sequencing revealed that GAL-8 C-terminal CRD binds to α_M_ integrin (also called CD11b) and pro-matrix metalloproteinase-9 (pro-MMP-9), while the N-terminal CRD binds to pro-MMP-9. A mutant GAL-8 lacking the N-terminal carbohydrate-binding activity (GAL-8R69H) retained pro-adhesive properties, while inactivation of C-terminal CRD (GAL-8R233H) abolished this function. Importantly, GAL-8R69H but not GAL-8R233H stimulated superoxide production in neutrophils (173). Besides, in pull-down assays with Jurkat T cell lysates, α_1_, α_3,_ and α_5_β_1_ integrins were the main GAL-8-interacting proteins. While α_5_ and β_1_ antibodies inhibited GAL-8-mediated cell adhesion by 60%, anti-α_1_ and -α_3_ antibodies suppressed about 30% of this effect (174). Lymphocyte function-associated antigen-1 (LFA-1), composed of α_L_ and β_2_ subunits, also proved to bind to GAL-8 in a carbohydrate-dependent manner, and this interaction was displaced by anti-GAL-8 autoantibodies isolated from patients with systemic lupus erythematosus (SLE). Moreover, in adhesion assays using peripheral mononuclear cells immobilized into ICAM-1, soluble recombinant GAL-8 inhibited cell binding, probably blocking the interaction between LFA-1 and ICAM-1 (175).

In platelet lysates subjected to GAL-8-affinity chromatography and mass spectrometry analysis, α_IIb_ subunit from the α_IIb_β_3_ integrin and glycoprotein (GP) Ib and V from GPIb-IX-V complexes, respectively, were the only membrane proteins identified that could eventually act as receptors for GAL-8 (176). To further characterize the possible role of α_IIb_β_3_ integrin and GPIb-IX-V as GAL-8 receptors, studies were performed using platelets from a patient with Glanzmann Thrombasthenia (GT), deficient in α_IIb_β_3_ integrin, or from a patient with Bernard Soulier (BS) syndrome whose platelets lack GPIb or platelets whose GPIb was cleaved by pre-treatment with trypsin. Consistently, a significant decrease of GAL-8 binding was observed in GT, BS, or trypsin-treated platelets. Due to the absence of α_IIb_β_3_ integrin, both collagen- and GAL-8-induced platelet aggregations were profoundly decreased in GT platelets. P-selectin exposure mediated by either collagen or GAL-8 was not affected despite the absence of α_IIb_β_3_, pointing out that α_IIb_β_3_ integrin is dispensable for GAL-8-induced platelet activation. Notably, platelet aggregation and P-selectin expression driven by GAL-8 exposure were almost absent in BS platelets. The mild aggregation response observed at the highest GAL-8 concentration employed in both GT and BS platelets was not inhibited by EDTA, indicating that it might be the result of an agglutination effect. These findings suggest that platelet GPIb, but not α_IIb_ integrin, was essential for GAL-8-dependent signaling, and thus represents a biologically relevant GAL-8 receptor (176).

In 1299 lung adenocarcinoma cells, GAL-8 selectively interacted with α_3_, α_6,_ and β_1_ integrins, as determined by immunoprecipitation and N-terminal sequencing (170). The addition of soluble GAL-8 to a suspension of 1299 cells markedly inhibited their adhesion to plates coated with laminin, fibronectin or the 120 kDa-proteolytic fragment of fibronectin (which includes its integrin-binding domain). Thus, soluble GAL-8 inhibited cell adhesion in a dose- and glycan-dependent fashion by interacting with cell surface integrins (170). Finally, in MDCK cells, α_5_ integrin, β_1_ integrin and EGFR were detected as putative ligands for GAL-8 in pull-down experiments, and GAL-8 interaction with α_5_β_1_ integrin triggered activation of the FAK/EGFR pathway (177).

### Podoplanin and VEGFR3

In lymphatic vasculature, GAL-8 interacts with PDPN at the surface of lymphatic endothelial cells (LECs) to induce adhesion and haptotaxis (178). VEGFR3 functions as a GAL-8-binding partner involved in lymphangiogenesis (179). Exogenous GAL-8, but not GAL-1, -3 and -7, markedly enhanced VEGF-C-induced LEC sprouting. This effect was five times higher in the presence of GAL-8 than that seen by VEGF-C alone. Moreover, in GAL-8-deficient mice, the extent of VEGF-C-induced lymphangiogenesis was significantly reduced (179). Podoplanin (PDPN) expressed in LECs interacted with GAL-8, but not GAL-1, -3 or -7, and the binding of PDPN to GAL-8 was carbohydrate-dependent: removal of α2-3-sialylated glycans by treatment with α2-3 neuraminidase abrogated PDPN interaction. PDPN knockdown not only inhibited GAL-8-induced LEC sprouting but also reduced VEGF-C-driven LEC sprouting substantially. Consistently, PDPN knockdown in LECs markedly reduced GAL-8- and VEGF-C-induced activation of AKT but not ERK1/2 (179). In summary, VEGF-C-induced lymphangiogenesis is significantly reduced in *Lgals8*^−/−^ and *Pdpn*^−/−^ mice, and GAL-8-induced lymphangiogenesis is attenuated in *Pdpn*^−/−^ mice. Interestingly, knockdown of VEGFR-3 did not affect GAL-8-mediated LEC sprouting, but inhibition of α_1_β_1_ and α_5_β_1_ integrins decreased both GAL-8- and VEGF-C-driven effects. Besides, PDPN co-immunoprecipitated with endogenous GAL-8 and specific integrins (α_1_, α_5_, α_v_, β_1_, but not α_9_ or β_3_), indicating that PDPN interacts with the endogenous lectin and the association between PDPN and these integrins was constitutive. Thus, lymphangiogenesis involves a GAL-8-dependent crosstalk among VEGF-C, PDPN, and integrin pathways (179).

Importantly, Bieniasz-Krzywiec and colleagues found PDPN-expressing macrophages in 4T1 breast cancer-associated cells, which localize in proximity to tumor lymphatics. In those tumors, PDPN was almost exclusively expressed in tumor-associated macrophages, but not in other tumor-infiltrating leukocytes. The interaction of PDPN and β1 integrin led to PDPN activation of β1 integrin. In fact, GAL-8 binding to PDPN on the macrophage surface induced the formation of a multicomplex with β1 integrin, thus promoting macrophage migration and adhesion to LECs. Interestingly, PDPN deletion in macrophages reduced tumor lymphangiogenesis and lymph invasion (180).

#### Activated leukocyte cell adhesion molecule (ALCAM or CD166)

In search for GAL-8-binding partners on bovine aortic endothelial cells (BAECs) by affinity chromatography coupled to mass spectrometric analysis, we identified ALCAM, a heavily glycosylated cell surface transmembrane immunoglobulin-like protein, as a GAL-8 putative ligand. Evaluation of ALCAM-GAL-8 interaction by SPR, showed an apparent *Kd* of 2 × 10^−6^ M, indicating specific binding. *In vitro* experiments in the presence or absence of anti-ALCAM antibodies showed that ALCAM mediates (at least in part) GAL-8 effects on endothelial cell migration and tubulogenesis (181).

We also found that endogenous ALCAM (CD166) is a GAL-8 ligand in human MDA-MB-231 breast cancer cells, showing a *Kd* of 3.19 × 10^−6^ M by SPR (182). Notably, ALCAM knockdown decreased *in vitro* cell binding and migration onto recombinant GAL-8. Moreover, glycoprofiling of endogenous ALCAM isolated from MDA-MB-231 cells revealed the presence of 34 structures including a major proportion of complex *N*-glycans, with prevalent bi- or tri-antennary structures. ALCAM complex *N*-glycans presented no sulfation and around 30% of sialylated structures. We further analyzed sialic acid linkage type by using a specific α2–3 neuraminidase and an α2–3,-6,-8,-9 neuraminidase A, demonstrating that mono-sialylated glycans are α2–3-sialylated, while the proportions of α(2–3) and α2–6-sialylated structures varied in di- and tri-sialylated structures. Tetra-siaylated structures were only represented by approximately 2.5% of the complete glycoprofile. An important proportion of the characterized *N*-glycans (45%) might be recognized by GAL-8 through interactions with α2–3-sialylated or neutral terminal *N*-acetyllactosamine residues. When digested with α2–3,-6,-8,-9 neuraminidase, the percentage of permissive structures considerably increased (63%). However, as exposure of glycan epitopes was dependent on glycoconjugate structure and abundance on the cell surface, not all permissive *N*-glycans described for ALCAM may be exposed or recognized by GAL-8 on MDA-MB-231 cells. As expected, desialylation increased MDA-MB-231 cell adhesion onto GAL-8-coated surfaces. Thus, a glycan-dependent effect of GAL-8, acting as a matricellular ligand on the adhesion and motility of ALCAM^+^ breast tumor cells, was confirmed (183).

Renard and coworkers validated the interaction between GAL-8 and CD166 (in HeLa cells, demonstrating that endocytic sites from which CD166 was taken up in an endophilin A3-dependent manner were driven by extracellular GAL-8. Thus, a clathrin-independent endocytic process mediated by CLICS and controlled by GAL-8 and endophilin A3 proved to be essential for down-modulation of the tumor marker CD166 at the cell surface, regulating adhesive and migratory properties of cancer cells (184).

#### CD45

CD45 isoforms were identified as putative GAL-8 receptors in mouse splenocyte extracts, which were initially subjected to a GAL-8 affinity column and eluted with lactose (185). The main protein bands were analyzed by MALDI-TOF after in-gel trypsin digestion, detecting several leukocyte surface glycoproteins, including CD45 and integrins. CD45R (also known as B220), found in B cells, together with the CD45RA present in naive T cells and the CD45RO isoform present in activated/memory T cells were identified as important GAL-8 partners. The reactivity of GAL-8 with the CD45R isoform on B cells was confirmed by performing MALDI assays in preparations of splenocytes from athymic *nude* mice. In addition, several surface markers from macrophages and neutrophils (such as sialoadhesin, macrophage mannose receptor, Mac-1, CD177, and MMP-9) were also found as putative GAL-8 receptors. Splenocyte proliferation was prevented by the addition of a CD45 phospho-tyrosine phosphatase (PTPase) inhibitor in the presence of GAL-8 concentrations ranging from 0.25 to 0.8 μM, demonstrating that GAL-8-CD45 interaction was involved in cellular proliferation. When GAL-8 was tested at higher concentrations (2 μM), the effect of the PTPase inhibitor on proliferation was severely reduced to 30%. Activation of the MAPK pathway by GAL-8 resulted in pERK1/2 increase in Jurkat T cells, which mediated T cell proliferation induced by this lectin; this effect was prevented by a CD45 PTPase inhibitor. These findings support the involvement of CD45 and MAPK pathways in GAL-8-induced T cell proliferation (185).

#### B cell receptor (BCR)

GAL-8 was also shown to interact with BCR. Pull-down experiments were conducted with murine spleen B cell extracts and glutathione-S-transferase (GST)-GAL-8-beads, followed by proteomic analysis. Interestingly, GST-Gal-8 formed a complex with the BCR, an interaction that was inhibited by lactose. GAL-8 barely affected SYK and ERK signaling, but it enhanced the phosphorylation of B cell linker protein (BLNK), Bruton’s tyrosine kinase (BTK), and AKT in the PI3K pathway. Furthermore, GAL-8 promoted the recruitment and secretion of lysosomes at the B cell synapse. Thus, GAL-8 facilitates synapse formation, lysosome secretion, and the proteolytic extraction of antigens. Collectively, these results indicate that, by directly interacting with BCR, GAL-8 increases B cell capacity to present antigens to helper T cells *in vivo* (186).

#### CD44

In synovial fluid cells from rheumatoid arthritis patients, GAL-8 was identified as a high-affinity ligand of CD44, showing special binding activity to CD44vRA, the splicing variant found in this fluid (187). The binding affinity of CD44vRA as determined by SPR analysis was the highest (*Kd*: 5.8 × 10^−9^ M) and was five- and 170-fold greater than that of CD44v3-10 (*Kd*: 2.7 × 10^−8^ M) and CD44s (*Kd*: 10 × 10^−6^ M), respectively. Moreover, the binding of GAL-8 to CD44 selectively activated JNK to promote CD44 signaling pathways. In fact, GAL-8 is involved in a ternary complex that includes soluble CD44 and fibrinogen. The binding of soluble fibrinogen and CD44 to GAL-8 prevented the pro-apoptotic activity of this lectin in inflammatory synoviocytes from rheumatoid arthritis patients, resulting in enhanced inflammatory responses. Thus, the severity of autoimmune inflammation might be influenced by several antagonistic factors that shift the balance toward stronger (*e.g.*, cell surface CD44vRA/FGF-2 or GAL-8/soluble CD44vRA/fibrinogen complexes) or weaker inflammatory responses (187).

## Cell surface GAL-9-binding partners

### T cell immunoglobulin and mucin domain-containing protein 3 (TIM-3), programmed cell death protein 1 (PD-1), and V-domain Ig-containing suppressor of T cell activation (VISTA)

TIM-3 is a type 1 cell-surface glycoprotein identified as a cell surface molecule expressed on CD4^+^ Th1 cells in mice (188, 189). In humans, it is expressed in T cells, NK cells, monocytes, and dendritic cells (189). GAL-9 was demonstrated to be a natural ligand of TIM-3; it binds to the N-terminal immunoglobulin variable (IgV) domain of TIM-3 through its two CRDs (190) (Table 5). GAL-9 binding to TIM-3 allows downstream signals that suppress TCR-mediated activation and promote T cell death (190).

Evidence suggested that the immunosuppressive effects driven by acute myeloid leukemia (AML) cells involve interactions between TIM-3 and GAL-9 through protein kinase C (PKC)/mTOR pathways (191). Interestingly, PKC activation occurred in a G-protein-coupled neuronal receptor latrophilin 1 (LPHN1)-dependent manner. Therefore, the LPHN1/PKC/mTOR/TIM-3/GAL-9 pathway is co-opted by human AML cells leading to decreased immune surveillance and promotion of disease progression (191, 192). TIM-3/GAL-9 autocrine loop was also demonstrated to be a critical mechanism for clonal dominancy and self-renewal of leukemic stem cells (193).

Primary breast tumors were also found to express significantly higher levels of GAL-9 and TIM-3, compared to healthy tissues of the same patients. Furthermore, as in AML cells, breast cancer cells showed colocalization of TIM-3 and GAL-9 and co-opted GAL-9 to prevent cytotoxic T-cell attack (194).

GAL-9 (but not GAL-1 or GAL-8) was also found to bind PD-1 in a glycan-dependent manner (Table 5). Notably, PD-1 ligand (PD-L1) and GAL-9 did not compete for the same binding site and, consequently, PD-1/PD-L1 neutralizing antibodies did not abrogate GAL-9/PD-1 interaction (193). Noteworthy, PD-1/TIM-3/GAL-9 lattices protected T cells from GAL-9/TIM-3-mediated apoptosis. Moreover, VISTA was also able to form heteromeric complexes with GAL-9 and TIM-3, promoting inhibition of granzyme B release by T cells (195).

### 4-1BB, DR3, CD44, CD40, and protein disulfide isomerase (PDI)

GAL-9 was also found to interact with 4-1BB co-stimulatory molecules on the surface of T cells, dendritic cells, and NK cells, mediating the generation of CD8^+^FoxP3^−^ Treg cells (196). Furthermore, in CD4^+^FoxP3^+^ Tregs, the interaction of GAL-9 with DR3, a member of the TNF/TNFR superfamily is crucial for the restoration of immune homeostasis during autoimmune inflammation (197). With regards to Treg cells, GAL-9 has been demonstrated to bind CD44 forming a complex with TGF-β receptor I (TGF-βRI), which enhances SMAD3 signaling and promotes inducible Treg cell differentiation (198). GAL-9 can also interact with CD40 and inhibit T-cell proliferation in a glycan-dependent, TIM-3-independent fashion (199). Strikingly, an additional receptor for GAL-9 on T cells is PDI, an enzyme that promotes disulfide bond formation. GAL-9 binding to PDI retained this enzyme on the cell surface and increased Th2 cell migration and HIV infection (200).

### CD45

Within the B cell compartment, GAL-9 is predominantly expressed on naive B cells where it suppresses BCR calcium flux in both B cell-intrinsic and extrinsic manner. In fact, this lectin has been demonstrated to bind CD45, inducing LYN-CD22-SHP-1 inhibitory signaling and moderate intracellular calcium levels, attenuating NFAT1 nuclear translocation, and B cell activation. Notably, I-branched *N*-glycans (or blood group I antigens) generated by the β1,6-*N*-acetylglucosaminyltransferase GCNT2 modulated binding of GAL-9 to naive, germinal center, and memory B cells, and the altered glycosylation of CD45 (*via* I-branching) was the predominant mechanism influencing GAL-9 binding (201). Moreover, GAL-9 altered the organization of CD45 and CD22 at the cell surface with regard to IgM-BCR, an event that correlated with BCR signaling suppression (202).

### CD206, Dectin-1 and IgE

In melanoma-associated M2-type macrophages, GAL-9 binds to CD206, increasing macrophage-dependent angiogenesis and worsening patient prognosis (203). Additionally, in experimental pancreatic adenocarcinoma, GAL-9 interacts with the C-type lectin Dectin-1 present on tumor-associated macrophages in a glycan-independent fashion. GAL-9 neutralization decreased tumor progression and restored T-cell activation (204).

GAL-9 also interacts with IgE and suppresses antibody-antigen complex formation, ameliorating symptoms of asthmatic reaction and passive cutaneous anaphylaxis in mouse models. Remarkably, inflammatory stimuli caused an increase in GAL-9 production by mast cells, generating an autocrine regulation of mast cell activity (205).

### Glucose transporter 2 (GLUT2)

A number of GAL-9 receptors have also been described in non-immune cell types. GLUT2 belongs to the structurally conserved GLUT protein family with 12 transmembrane domains and one *N*-glycosylation site. It is expressed in the liver, kidney, intestine, pancreatic islet β cells, and central nervous system. In pancreatic β cells, GLUT2 is required for glucose-stimulated insulin secretion (206, 207). Interestingly, fluorescence deconvolution microscopy analysis revealed the colocalization of GLUT2 and GAL-9 in pancreatic β cells isolated from wild-type mice tissues. However, colocalization was significantly reduced in pancreatic β cells derived from GlcNAcT-IV glycosyltransferase (MGAT4a/GnT-4a)-deficient mice, as reflected by diminished GLUT2 cell-surface expression. Cell-surface protein cross-linking and co-immunoprecipitation studies demonstrated that GLUT2 and GAL-9 are in close proximity to the cell surface (Table 5). These findings suggested that GAL-9 interacts with GnT-4a (MGAT4a)-dependent glycosylated GLUT2 in pancreatic β cells which may reduce GLUT2 endocytosis thus sustaining glucose-stimulated insulin secretion, an effect that would be relevant in type 2 diabetes (208).

### CD146

The CD146 adhesion molecule is present on the blood–brain barrier (BBB), but not on immune cells, in a mouse model of experimental cerebral malaria (eCM) (209). Endothelial CD146, upregulated during eCM development, was demonstrated to sequestrate infected red blood cells and proinflammatory lymphocytes in central nervous system vessels, promoting disruption of BBB integrity. Tandem-mass spectrometry studies showed that GAL-9 was a potential ligand of CD146 in BBB endothelial cells (BBBECs) stably transfected with CD146-Flag plasmid and T cells isolated from healthy or eCM mice. These results were confirmed by co-immunoprecipitation, pull-down with blocking antibodies and ELISA experiments. Hence, the GAL-9-CD146 interaction might contribute, at least in part, to the adhesion of BBBECs to T cells (209).

## Extracellular matrix glycoproteins: common ligands of different galectins

Galectins can bind to several extracellular matrix (ECM) molecules, such as laminin, fibronectin, thrombospondin, vitronectin, and glycosaminoglycans, through carbohydrate-protein interactions (72). The functional outcome of these interactions will depend on the cell type and its activation status as well as the relative expression of these matricellular proteins.

Many cellular processes, such as adhesion, proliferation, and differentiation, involve interactions between different cell types and laminin (210, 211). This glycoprotein can interact with integrins and gangliosides on cell membranes or with other ECM molecules such as type IV collagen and heparan sulfate, among others. Both laminin and fibronectin are highly *N*-glycosylated proteins and constitute the main GAL-1 receptors in the extracellular milieu (72, 210, 212). On laminin-coated surfaces, GAL-1 modulated vascular smooth muscle cell (SMC) migration whereas it inhibited cell migration onto fibronectin. Moreover, since GAL-1 binds to several proteins in SMC extracts, including laminin and α_1_β_1_ integrin, this lectin has been proposed to modulate SMC attachment, spreading, and migration *via* interactions with ECM proteins and α_1_β_1_ integrin (81).

Other ECM proteins such as thrombospondin, osteopontin, and vitronectin were also identified as potential ligands for GAL-1 in vascular SMCs. In addition, GAL-1 was described to interact with both heparan sulfate and chondroitin; these glycosaminoglycans were shown to reduce the binding of GAL-1 to ECM proteins. These findings denote that GAL-1 may be involved in vascular ECM assembly by modulating the incorporation of some ECM components, suggesting that it might be relevant during wound healing and tissue matrix remodeling (213, 214).

Likewise, GAL-3 binds to laminin, collagen, and fibronectin. It has been demonstrated that GAL-3 acts as a bridge between human neutrophils and laminin, promoting adhesion in a glycan-dependent and cation-independent manner. These findings imply that GAL-3 might be involved in facilitating neutrophil movement across the basement membrane at inflammation sites (215). In addition, GAL-3 was found to augment β_1_ integrin-mediated cell adhesion to laminin, fibronectin, and type 1-collagen in epithelial cells, as well as cell migration (216). GAL-3 also increased corneal epithelial cell adhesion to ECM components in Bowman's membrane (types 1 and V collagen) and in the basement membrane (type IV collagen, fibronectin, laminin-5), and to ECM cellular ligands (α_1_β_1_, α_5_β_1_, α_3_β_1_ integrins). These results suggest the involvement of GAL-3 in ECM association and cell migration over wounded corneal surfaces (217).

GAL-8 also binds to ECM proteins such as fibronectin due to specific protein-saccharide interactions (218). Serum proteins were incubated with immobilized recombinant GAL-8 and the bound proteins were studied after selective elution with lactose; the identity of fibronectin was confirmed by blotting lactose-eluted proteins with specific antibodies. Peptide sequencing by matrix-assisted laser desorption ionization (MALDI) analysis revealed eight peptides derived from fibronectin corresponding to 6.6% of the total protein mass. Most importantly, a higher number of HeLa cells adhered to GAL-8-coated plates when they were pre-coated with fibronectin, indicating that GAL-8 and fibronectin effects are partially additive (218).

Reticker-Flynn and coworkers developed an ECM microarray platform to assay combinations of ECM molecules for cell attachment in a mouse model of lung adenocarcinoma (219). Fibronectin/GAL-8 combinations were tested in adhesion assays with cell lines derived from primary lung tumors *versus* cell lines derived from their metastases. Identification of metastasis-associated ECM molecules revealed higher tumor progression when fibronectin was assayed in combination with GAL-8, GAL-3, or laminin. When lymph node metastases were analyzed, they showed high expression of the metastasis-associated molecules fibronectin, laminin, GAL-8, and GAL-3. In conclusion, the combination of GAL-8 with fibronectin is strongly associated with metastatic progression in lung adenocarcinoma (219).

Moreover, GAL-9 was demonstrated to bind to human laminin, fibronectin, and thrombospondin, whereas no binding was observed with other ECM components, including collagens, elastin, and vitronectin (220). In conclusion, galectins may exert their regulatory actions according to the composition of the ECM microenvironment in a particular cellular context.

## Different galectins bind to the same cell surface receptor: biological relevance

In this section, we discuss the biological relevance of key processes associated with galectin-glycan recognition, particularly in tumor and inflammatory microenvironments. We illustrate particular cases where the same receptors are recognized by more than one galectin, emphasizing cell surface ligands and those associated with the ECM.

### TF and MUC glycoepitopes

**Figure 6:**
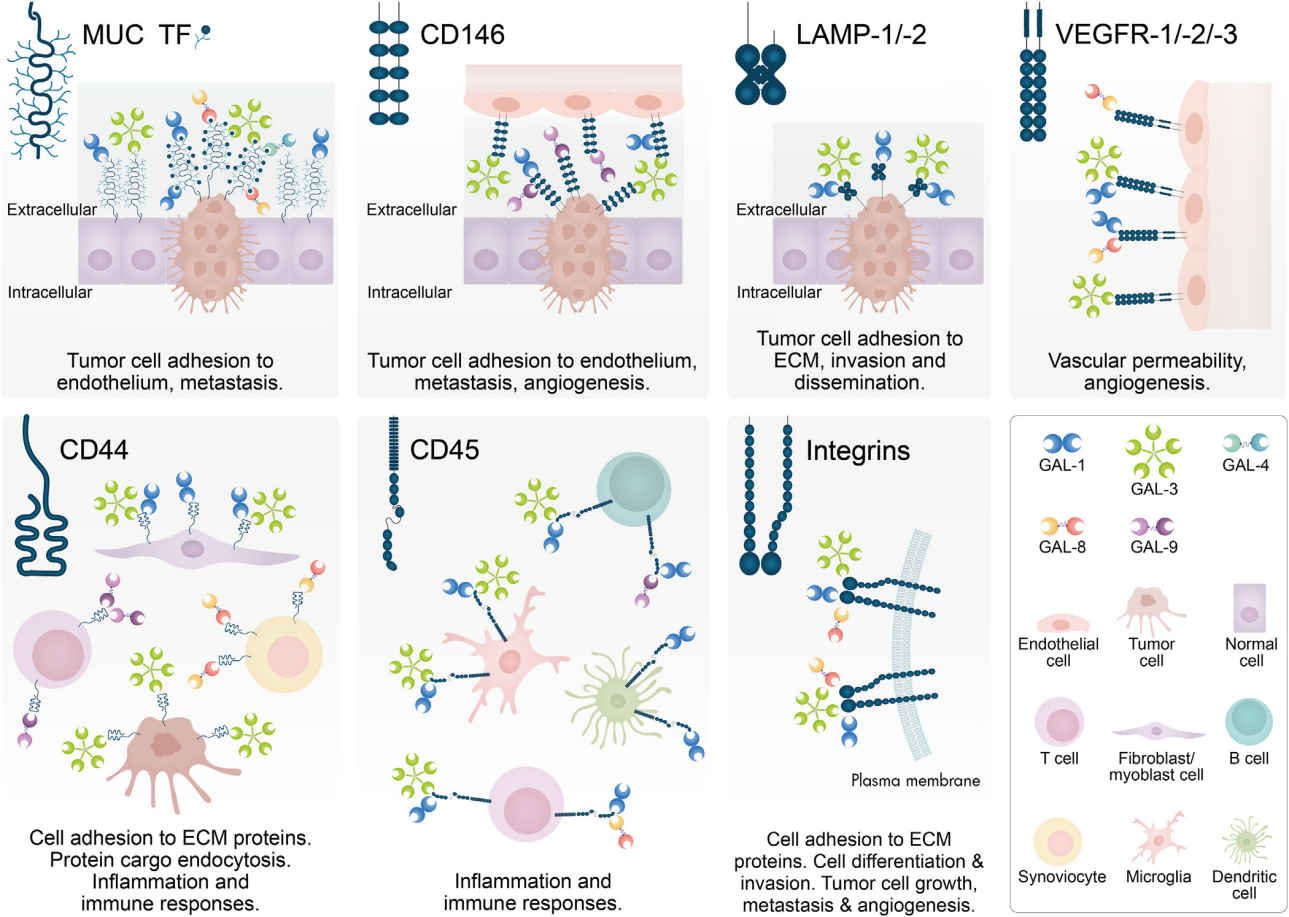
**Various glycosylated receptors are recognized by more than one galectin on different cell types.** TF/MUC is a receptor for GAL-1, GAL-3, GAL-4, and GAL-8; CD146 is a glycosylated receptor for GAL-1, GAL-3, and GAL-9; LAMP-1/-2 is engaged by GAL-1 and GAL-3. *N*-glycan-dependent intracellular interaction between GAL-9 in the lysosomal lumen and LAMP-2 in gut epithelial cells (230) is not shown. VEGFR-1/-2/-3 and integrins are receptors for GAL-1, GAL-3 and GAL-8; CD44 and CD45 are receptors for GAL-1, GAL-3, GAL-8 and GAL-9. As a result of their glycan dependence or independence, the multivalent nature of these interactions, and the underlying signaling pathways, galectins play key roles in a wide range of biological programs.

Adhesion of human breast carcinoma cells to the endothelium has been shown to be mediated by GAL-1 and GAL-3 through interaction with TF antigen (53, 128). Particularly, recombinant GAL-3, at concentrations similar to those found in the circulation of patients with metastatic cancer, significantly increased the adhesion of breast and colon cancer cells to the endothelium as a consequence of its interaction with TF expressed on MUC1, causing a redistribution of this glycoprotein on the cancer cell surface (125). Interestingly, co-incubation with anti-E-selectin or anti-CD44H antibody inhibited GAL-3-MUC1-mediated cell adhesion to HUVECs (125). In line with these findings, the interaction of cell-surface MUC1 with GAL-3 at pathologically relevant concentrations reduced the protective effects of MUC1 on cancer cell adhesion, transendothelial invasion, and metastasis. GAL-3 effects were mediated by MUC1 cell surface clustering and the consequent exposure of cell-surface CD44 and endothelial-E-selectin ligands (221). The expression of TF antigen in MUC1 was also demonstrated to be important in the regulation of GAL-4- and GAL-8-mediated cancer-endothelium adhesion (165). Therefore, higher levels of galectins in sera from cancer patients and increased expression of MUC1 and TF antigen-associated MUC1 by cancer cells might enhance molecular interactions between circulating galectins and TF/MUC1, promoting tumor cell adhesion to vascular endothelium and metastasis (222) (Fig. 6).

GAL-1 was also described to bind the TF glycotype on trophoblast cells and to inhibit BeWo chorionic carcinoma cell proliferation (54). In fact, the placenta resembles the invasion of malignant tumors (223). During the first trimester of pregnancy, trophoblast cells from the fetus invade the maternal decidua. GAL-1, localized at the border between fetal trophoblast and maternal stroma, may promote trophoblast cell invasion *via* interaction with TF antigen, and suppress maternal T cells within the decidua, contributing to the maintenance of pregnancy (54). Additionally, TF epitope and MUC1 co-expression and simultaneous binding of GAL-1 to TF antigen on human endometrial epithelial apical surface tissue in the early secretory phase, on human oocytes and irregularly fertilized oocytes led to the suggestion that GAL-1 might mediate trophectoderm binding to the endometrium within the window of implantation (55). Furthermore, GAL-3 binding to MUC1 and MUC16 at the apical membrane of corneal epithelium is critical for the maintenance of the epithelial barrier and protection against herpes simplex virus (HSV)-1 infection (131, 132). Therefore, the interaction between galectins and TF antigen-associated mucins may also modulate important physiological processes, such as implantation, pregnancy maintenance, and protection from viral infection.

### CD146

The association of the highly glycosylated adhesion molecule CD146 with GAL-1 was demonstrated to protect the endothelium from GAL-1-induced apoptosis, suggesting that CD146 could serve as a decoy receptor for this lectin, preventing its function (48). In addition, given that GAL-1 activates VEGFR-2 and NRP-1 signaling and modulates the migration of vascular endothelial cells (37) and that CD146 is a co-receptor for VEGFR-2 in tumor angiogenesis (224), CD146 cross-linking by GAL-1 might play a role in VEGFR-2 signaling (224). Remarkably, GAL-1 binding to *N*-glycans on VEGFR-2 and activation of VEGF-like signaling in anti-VEGF-A refractory tumors fostered tumor progression and angiogenesis (45). Thus, the GAL-1/CD146/VEGFR-2 axis critically regulates angiogenesis during tumor progression.

CD146 is also recognized by GAL-3. Interestingly, silencing CD146 expression completely abolished GAL-3-induced secretion of IL-6 and G-CSF by endothelial cells. Thus, it was proposed that circulating GAL-3 could interact with CD146 on endothelial cells and induce secretion of metastasis-promoting cytokines (120, 121). Moreover, during eCM development, CD146 was found to be upregulated in BBBECs. As GAL-9 levels were reported to be increased in malaria patients, it was suggested that CD146 may play an important role in eCM development through its interaction with GAL-9 (209).

The biological relevance of galectin-CD146 interactions on other cell types/tissues has also been demostrated. As an example, higher CD146 was detected during preeclampsia which was described to interact with GAL-1 to regulate trophoblast migration through VEGFR-2 (225). Furthermore, CD146 was demonstrated to play a critical role in obesity. It was demonstrated that CD146 is upregulated in the white adipose tissue of obese mice and humans, where it mediates the detrimental effects of high-fat diet (HFD)-induced obesity, such as insulin resistance, fatty liver, and inflammation. Besides, through its interaction with both GAL-1 and ANGPTL2, CD146 promoted adipogenesis and lipid accumulation, and suppressed energy expenditure (49).

Therefore, in cancer settings, where tumors elude immune responses and induce angiogenesis or in pathogen-driven inflammatory processes in which GAL-1, GAL-3 or GAL-9 levels are increased, endothelial CD146 can be recognized by one or more of these galectins (Fig. 6). These interactions may critically regulate cancer progression, metastasis, tumor-related angiogenesis, and pathogen invasion. Moreover, galectins might also regulate critical physiological and pathological processes, including pregnancy, preeclampsia, lipid metabolism, and obesity through binding to CD146.

### LAMP-1 and LAMP-2

In human metastasizing melanoma, fibrosarcoma, ovarian, and colon carcinoma cells, LAMP-1 and LAMP-2 were found to serve as GAL-1- and GAL-3-binding partners (67, 68, 226). These cell surface receptors mediate ovarian carcinoma cell adhesion to ECM induced by GAL-1 (67). By interacting with poly-LacNAc structures on LAMP-1, GAL-3 induced the expression of MMP9 in a p38 MAPK-dependent manner, revealing a possible mechanism for melanoma cell invasion and metastasis (227). Enhanced expression of LAMP-1 and LAMP-2 on the tumor cell surface, and/or increased number of poly-LacNAc residues on LAMPs, have been shown to stimulate association with galectins. As a consequence of these interactions, tumor cell adhesion to ECM, basement membrane, and endothelium, as well as tumor cell invasion and dissemination may be promoted (Fig. 6).

With regard to the immune system, leukocyte surface LAMP-1 and LAMP-2 participate in cell-cell adhesion and in protecting the plasma membrane from enzymes released after leukocyte degranulation (64, 228). GAL-1 colocalized with LAMP-1 and LAMP-2 in cytotoxic granules and modulated CTL killing activity, retaining Fas ligand (FasL) on the CTL surface and preventing its internalization. Remarkably, CTLs from GAL-1-deficient mice showed decreased capacity to eliminate antigen-specific cell targets *in vivo* (69). These findings revealed a central role of GAL-1 in preventing receptor endocytosis, thus amplifying FasL-mediated killing activity.

Recently, using CRISPR-Cas9 technology, knocking out GAL-9 in human hepatocarcinoma cells revealed the role of this lectin in ubiquitination in response to lysosomal damage with Leu-Leu-OMe (LLOMe) (229). Proteomic proximity analysis of GAL-9 during lysosomal damage revealed that this galectin interacts with LAMP-1 and LAMP-2 (229). Interestingly, *N*-glycan-dependent interaction between GAL-9 in the lysosomal lumen and LAMP-2 has also been demonstrated by co-immunoprecipitation in gut epithelial cells (230). The use of LAMP-1/2 and GAL-9 knockout CMT93 mouse rectum epithelial cells, and mutant proteins of each *N*-glycosylation site in LAMP-2 led to the conclusion that, through binding to glycosylated moieties attached to Asn^175^ residue in LAMP-2, GAL-9 maintains lysosome function to facilitate autophagy and prevent ER stress-associated cell death. Silencing key enzymes involved in the synthesis of poly-LacNAc confirmed that the interaction of GAL-9 with poly-LacNAc was critical for lysosome function. Besides, it was demonstrated that GAL-9 regulates lysosome function in acinar cells to prevent pancreatitis. Collectively, these findings unveiled the critical role of GAL-9 in controlling the function of lysosomes and autophagy to avoid cell damage in the pancreas and intestine (230). Thus, glycosylated LAMPs may function both as cell surface and intracellular binding partners of galectins.

### CEA

CEA was described as an endogenous receptor for GAL-1, GAL-3, and GAL-4 in human colon carcinoma cells and in colon carcinoma liver metastasis (68, 72, 126, 127, 163). Knocking-down GAL-3 in colorectal carcinoma cells did not affect CEA expression; however, it suppressed cell migration induced by CEA. Importantly, CEA colocalized with GAL-3 in colorectal carcinoma patient tissues, and high serum CEA and GAL-3 levels correlated with advanced stage and poor survival in colorectal carcinoma patients. These results highlight the critical role of the GAL-3/CEA axis in colorectal cancer metastasis (127).

CEA is also a biologically important ligand for GAL-4 in human colon adenocarcinoma cells (163). GAL-4 was localized in patches with CEA, SB1a, and GM1 on CCK-81 and LS174T cells. In adhesion assays, binding of LS174T cells to GAL-4-coated plates was dose-dependent and inhibited by anti-SB1a, suggesting that GAL-4 binds to 3-*O*-sulfated glycosphingolipids. When plates pre-coated with GAL-4 were sequentially incubated with different CEA concentrations, LS174T cell binding to this lectin was inhibited, suggesting that robust interactions between GAL-4 with both SB1a and CEA are required to promote colon adenocarcinoma cell adhesion (163).These results underlie the relevance of galectins-CEA interactions in promoting colon cancer metastasis.

### Integrins

As a consequence of GAL-1 interaction with integrins, many important biological effects have been reported. For instance, GAL-1 modulated SMC attachment, spreading, and migration *via* interactions with α_1_β_1_ and α_7_β_1_integrins and ECM proteins (81, 82). GAL-1 and α_1_β_1_ integrin interaction also stimulated trophoblast cell adhesion and migration and modulated trophoblast integration into endothelial cell networks (83, 231). In human breast cancer cell lines, the interaction between GAL-1 and β_1_ integrin triggered the FAK/c-Src/ERK/STAT3/survivin pathway and promoted resistance to the chemotherapeutic drug doxorubicin (84). In this regard, α_5_β_1_ integrin expression, inhibition of Ras-MEK-ERK signaling pathway, and accumulation of cyclin-dependent kinase inhibitors p21 and p27 mediated GAL-1-carcinoma cell growth inhibition (232). Importantly, GAL-1 binding to α_5_β_1_ integrin, modulated by the cell surface sialylation status, also induced carcinoma cell anoikis (233). Moreover, antibody-mediated blockade demonstrated the involvement of α_1_, α_2_, α_3_, α_v_, and β_1_ integrins as critical mediators of the proadhesive effects of GAL-1 on HepG2 liver cancer cells (234). β_1_ integrin binding and activation of Hedgehog (Hh)/Gli1 signaling pathway were also found to be necessary for GAL-1-induced migration, invasion, and EMT of gastric cancer cells (235). Interestingly, the increased receptor for activated C-kinase 1 (RACK1) levels enhanced cervical cancer cell invasion and EMT, and promoted lymphangiogenesis and metastasis in a GAL-1- and β_1_ integrin-dependent manner (236).

Remarkably, stimulation of platelets by GAL-1 resulted in the activation of the major signaling pathways involved in the integrin outside-in signaling, including phosphorylation of the β_3_ cytoplasmic tail and activation of tyrosine kinases SYK and AKT as well as PLCγ2 (85). In addition, studies with blocking antibodies or α_IIb_β_3_-deficient platelets from patients with Glanzmann’s thrombasthenia syndrome revealed the relevance of GAL-1 in hemostasis and thrombosis (85).

Integrins were also identified as receptors of GAL-3 on endothelial cells. GAL-3-induced α_v_β_3_ integrin clustering promoted FAK activation and VEGF- and basic fibroblast growth factor (bFGF)-mediated angiogenesis on HUVECs. β1,6GlcNAc-branched *N*-glycans on α_v_β_3_-integrin were demonstrated to be critical for GAL-3 interaction with the endothelial cell surface (112).

Several integrins can bind to GAL-8 in different cell types. For example, α_5_β_1_ and α_v_β_1_integrins were described as major receptors of GAL-8 in trabecular meshwork (TM) cells in the eyes. GAL-8 modulated TM cell adhesion and spreading, at least in part, by interacting with α2-3-sialylated glycans on β_1_ integrins (237). GAL-8 also interacted with α_3_, α_6,_ and β_1_integrins in 1299 lung adenocarcinoma cells, and by modulating integrin interactions with the ECM, GAL-8 regulated cell adhesion and survival (170). In Jurkat T cells, α_1_, α_3_, and α_5_β_1_ integrins were the main GAL-8-interacting proteins. Besides, SLE patients generated GAL-8 autoantibodies that hindered GAL-8 binding to integrins and cell adhesion. Thus, GAL-8 could modulate a wide range of immune cell processes during autoimmune disorders (174).

Collectively, these findings indicate that galectins can establish multivalent interactions with glycosylated cell surface integrins (Fig. 6), cluster these molecules, and trigger signaling pathways that modulate skeletal muscle and vascular SMC differentiation, trophoblast cell invasion, cell adhesion to ECM proteins, platelet activation, tumor cell growth, EMT, metastasis, angiogenesis, and autoimmune responses.

### CD44

Different members of the galectin family bind CD44, a cell adhesion molecule and receptor for hyaluronan (Fig. 6). CD44 also acts as a protein cargo during clathrin-independent endocytosis in CLICs (117). GAL-1 has been described as a mediator of myogenic differentiation through binding to native CD44 in a glycan-mediated manner (100). Furthermore, GAL-3 has been shown to interact with *N*-glycosylated branches of CD44 and GAL-3 together with glycosphingolipids were required for the endocytosis of CD44 and β_1_ integrin. Thus, GAL-3 may function as an endocytic adaptor that promps clathrin-independent endocytic pit biogenesis by linking glycosylated cargo proteins and glycosphingolipids in a well-defined region of the plasma membrane (145). CD44 was also found to serve as a ligand for GAL-8 in synoviocytes from rheumatoid arthritis patients (187). In addition, GAL-9 directly interacts with CD44, which formed a complex with TGF-βRII, activating SMAD3, and increasing Treg cell stability and function (198). Notably, a sialyllactosaminyl-decorated CD44s glycovariant has been found to be a conserved feature of human mesenchymal stem cells (hMSCs) derived from adipose tissue and bone marrow (238). Whether this glyco-signature modulates galectin binding to hMSCs and modulates its functional activity remains to be investigated.

Thus, through interaction with CD44, galectins may modulate cell adhesion to ECM, endocytosis, inflammation and immunomodulation (Fig. 6).

### VEGFRs

VEGFRs, including VEGFR-1, VEGFR-2, and VEGFR-3, are key glycosylated receptors that mediate angiogenesis and lymphangiogenesis (239). As a receptor for VEGF, NRP1 forms complexes with VEGFR-1 and/or VEGFR-2, enhancing the binding of VEGF to VEGFRs and promoting VEGF-mediated angiogenesis and tumorigenesis (32, 33). Binding to the NRP1/VEGFR-1 complex coupled to activation of the AKT/Rho A signaling pathway was required for GAL-1-induced HUVEC permeability (44). Interestingly, GAL-1 was demonstrated to directly interact with NRP1, enhancing VEGFR-2 phosphorylation and stimulating the activation of the MAPK stress-activated protein kinase-1/c-Jun NH2-terminal kinase (SAPK1/JNK), thus promoting endothelial cell migration (37). Besides, the binding of GAL-1 to *N*-glycans on VEGFR-2 has been shown to modulate the cell surface residency of this receptor and its internalization. Moreover, GAL-1 binding to VEGFR-2 and activation of VEGF-like signaling in anti-VEGF-refractory tumors promoted compensatory angiogenesis and tumor progression (45). This effect was recently confirmed in clinically-relevant settings, demonstrating that GAL-1 present in sera from anti-VEGF (bevacizumab)-treated patients promotes VEGFR-2 phosphorylation and angiogenesis (240).

The binding of GAL-3 to *N*-glycans on VEGFR-2 was also demonstrated. Through this interaction, GAL-3 promoted VEGFR-2 phosphorylation and regulation of receptor internalization in human endothelial cells (112). The pro-angiogenic effect of GAL-3 and the role of *N*-glycan branching in corneal neovascularization were demonstrated using mice lacking GAL-3 or MGAT5, respectively (112). Furthermore, the addition of recombinant GAL-1 or GAL-3 to EA.hy926 endothelial cells induced phosphorylation of both VEGFR-1 and VEGFR-2 and tube formation (43).

Similar to VEGF-C, GAL-8 promoted lymphangiogenesis in two different mouse models. Strikingly, VEGFR-3 was demonstrated to function as a GAL-8-binding protein, which was clustered by this lectin and retained on the surface of LECs. However, VEGFR-3 knockdown did not inhibit GAL-8-induced sprouting. It was proposed that GAL-8 displays a unique dual-faceted mechanism of action to promote lymphangiogenesis, where its interactions between lymphangiogenic integrins (α_1_β_1_/α_5_β_1_) and PDPN are sufficient to activate integrins and trigger the lymphangiogenesis process without involvement of VEGFR-3. However, in the presence of VEGF-C/VEGFR-3, PDPN/GAL-8-integrin interactions substantially enhance lymphoangiogenesis by potentiating VEGF-C/VEGFR-3 signaling (179).

In conclusion, through binding to *N*-glycans on VEGFR-1, -2, and -3, galectins can induce receptor-tyrosine kinase activation and phosphorylation, thus promoting vascular permeability, tumor-related angiogenesis and/or lymphangiogenesis (Fig. 6). Future studies are required to evaluate the coordinated effect of different galectins in health and disease.

### CD45

The interaction between GAL-1 and the CD45 tyrosine phosphatase has been shown to contribute to the suppression of microglial cell activation and restoration of neuronal integrity through *O*-glycan-dependent mechanisms (103), whereas, on T cells, this interaction contributed to the induction of cell death programs (104). Remarkably, T helper signals increased CD45 phosphatase activity on B lymphoblastoid cells by amplifying surface binding of GAL-1 (109). Additionally, CD45 contributed to GAL-3-induced T-cell death (104), whereas in large B-cell lymphoma cells GAL-3/CD45 interaction modulated apoptosis resistance (155). Moreover, CD45 and MAPK signaling pathways have been shown to be critical in GAL-8-induced T-cell proliferation (185). Finally, through binding to CD45, GAL-9 inhibited activation of B cells (201, 202). Thus, galectins are critical regulators of both lymphoid and myeloid cell programs through glycan-dependent interactions with CD45 and modulation of its phosphatase activity (Fig. 6).

## Conclusions and future perspectives

Here we review the multiple galectin receptors present in different cell types and their most relevant functions, focusing on the mechanistic bases of galectin-glycan interactions in diverse physiologic and pathologic settings.

The evidence published over the past years revealed that different members of the galectin family can bind to the same receptors, depending on the specific cell type and the pathophysiological condition. This complex interplay can trigger and/or modulate identical or different signaling pathways, leading to a variety of cellular responses. Despite the intricacy of this scenario, we may conclude that galectins can interact with three types of receptors on cell surfaces. The first type includes matricellular adhesion molecules such as integrins, TF/MUC, CD44, CD146, and CD166, which play key roles in cell-cell and cell-ECM interactions. The second involves glycosylated ligands which function as enzyme-linked receptors, either associated with an enzyme or possessing enzymatic activity themselves, such as RTKs and CD45. Through these interactions, galectins have been shown to modulate cell surface residency and endocytosis of various glycosylated receptors. As a result, galectin-ligand interactions have the potential to amplify or disrupt cell-cell communication by influencing endocytosis, trafficking, and signaling of canonical receptors. Finally, the third group of receptors involves those regarded as immune checkpoints, like CTLA-4, TIM-3, LAG-3, and PD-1.

There are still several unanswered questions regarding interactions between galectins and their binding partners: Is the expression of galectins and their ligands linked by common signaling pathways? Interestingly, while both GAL-1 and NRP1 are upregulated in tumors from patients resistant to PLX-4720, an inhibitor of the proto-oncogene BRAF, silencing GAL-1 expression in melanoma cells significantly decreased NRP1 protein levels. This effect suggests a positive loop mediated by autocrine ligand production (241). Furthermore, GAL-1 and NRP1 pharmacological inhibitors (OTX-008 and EG00229, respectively) decreased refractoriness to BRAF-targeted therapy in melanoma cells (241). Therefore, deciphering the mechanisms involved in galectin-ligand interactions and their regulated expression may be relevant for understanding resistance or refractoriness to single or combination therapies (29, 242) and their association with other aberrant glycosylation patterns observed during tumor progression (243).

While certain galectin ligands have been known for years and studied extensively, new technologies such as mass spectrometry, microscale thermophoresis, and live cell proximity labeling are currently being employed to discover additional ligands and analyze their direct binding to galectins. However, the biological consequences of these interactions have yet to be investigated.

In conclusion, the versatility of the biological functions displayed by galectins is a result of their relative abundance, diversity, ligand promiscuity, and multiplicity of cellular targets. Therefore, identifying the various galectin receptors, understanding the molecular basis of this promiscuity, and characterizing the biochemical nature of these interactions, particularly their glycan dependency, might contribute to the design of novel galectin-based therapeutic strategies targeting a broad range of diseases, including cancer, chronic inflammation, autoimmune diseases, metabolic disorders, fibrosis, and neurodegeneration.

## Conflict of interest

M. F. T., M. T. E., A. G. B., L. S. and M. V. E. declare that they have no conflict of interest regarding the content of this article. G. A. R. reports a relationship with Galtec SAS that includes equities.
